# Effect of the Min System on Timing of Cell Division in *Escherichia coli*

**DOI:** 10.1371/journal.pone.0103863

**Published:** 2014-08-04

**Authors:** Shuxin Jia, Daniela Keilberg, Edina Hot, Martin Thanbichler, Lotte Søgaard-Andersen, Peter Lenz

**Affiliations:** 1 Department of Physics, Philipps-Universität Marburg, Marburg, Germany; 2 LOEWE Center for Synthetic Microbiology, Philipps-Universität Marburg, Marburg, Germany; 3 Max Planck Institute for Terrestrial Microbiology, Marburg, Germany; 4 Department of Biology, Philipps-Universität Marburg, Marburg, Germany; Indian Institute of Science, India

## Abstract

In *Escherichia coli* the Min protein system plays an important role in positioning the division site. We show that this system also has an effect on timing of cell division. We do this in a quantitative way by measuring the cell division waiting time (defined as time difference between appearance of a division site and the division event) and the Z-ring existence time. Both quantities are found to be different in WT and cells without functional Min system. We develop a series of theoretical models whose predictions are compared with the experimental findings. Continuous improvement leads to a final model that is able to explain all relevant experimental observations. In particular, it shows that the chromosome segregation defect caused by the absence of Min proteins has an important influence on timing of cell division. Our results indicate that the Min system affects the septum formation rate. In the absence of the Min proteins this rate is reduced, leading to the observed strongly randomized cell division events and the longer division waiting times.

## Introduction

Living in ever-changing environments bacteria are frequently forced to adjust internal processes to external conditions. Molecularly this is done by signal transduction pathways that sense external or internal signals, and generate an output response from the information encoded by these signals. In many instances, these pathways produce an oscillatory response in which the output varies over time in a recurrent manner. In general terms, three parts are essential to produce such an oscillatory response: an input pathway, an output pathway and an oscillator [1]. The input pathway adjusts the behavior of the oscillator to internal or external signals such as light, temperature or nutrition status. In this way it changes, e.g., the phase or the frequency of the oscillation. The oscillator itself (which is the main part of the system) uses some biochemical machinery to generate an oscillatory output. The output pathway then translates the behavior of the oscillator into a readable downstream signal [1]. The interaction between the input and output pathways and the oscillator can occur at different levels, for example by regulation of transcription, translation or at the post-translation level [2]–[4].

Generally, oscillators can be classified into two types: temporal oscillators and spatial oscillators [5]. Temporal oscillators determine when specific cellular events happen while spatial oscillators determine where they happen.

One way to implement temporal oscillations is to make the concentration of active proteins temporally varying throughout the entire cell. Two fundamental examples of temporal oscillators in bacteria are the circadian oscillator and the cell cycle oscillator. A circadian oscillator allows cells to adapt cellular activities to the changing conditions during the 24 hours diurnal period [6], [7]. The cell cycle oscillator, on the other hand, ensures the correct order of fundamental processes such as chromosome replication, chromosome segregation and cell division, and couples these to cell growth [8]–[10].

For our study it is important to take into account that the cell cycle consists of two independent cycles, namely the cycle of mass duplication and the cycle of chromosome replication [11], [12]. Both cycles have to be finished before cell division can take place [13]. The time between birth and subsequent division of a single cell is therefore typically limited either by the time needed until two completely replicated DNA strands have segregated or the time needed to reach division mass. However, despite considerable efforts it is not known how these two cycles are coordinated. The seminal work of Cooper and Helmstetter showed that there is a macroscopic relation between cell mass and initiation of DNA replication [14], [15]. But the molecular regulation that gives rise to this relation remains unclear [16]–[23]. Given these difficulties it is not surprising that only very little is known about the mechanisms that trigger cell division after the two cycles are completed [12].

While temporal oscillators typically regulate the temporal order of cellular events connected to cell growth and division, spatial oscillators are involved in positioning and localization of cellular components. To implement spatial oscillations the spatial distribution of proteins in the cell needs to be dynamically changing. The oscillation in the localization gives rise to a time-dependent spatial pattern. For example, the establishment of the correct cell polarity during A-motility in *Myxococcus xanthus* is the outcome of an spatial oscillator consisting of the proteins MglA and MglB and the Frz system [24], [25]. The plasmid segregation oscillator (the *parS*-ParA-ParB system) pulls plasmids back and forth in this way guaranteeing that plasmids are equally distributed in the daughter cells after division [26], [27]. A similar system is responsible for chromosome segregation in many bacteria [28]–[30].

Among spatial oscillators the Min system is one of the best studied examples [31]. It consists of the proteins MinC, MinD and MinE. In *E. coli* these proteins oscillate from pole to pole with a period of about 1-2 minutes [32]–[36]. As output of the spatial oscillations the Z-ring formed by FtsZ is positioned at mid-cell [37]–[40]. From many experimental and theoretical studies the following pictures has emerged on how these oscillations are implemented molecularly: MinC is inhibitor of Z-ring formation by FtsZ [41]–[43]. Thus, the Z-ring can only form at membrane positions with low MinC concentrations. MinC forms a complex with MinD [44], [45] and thus follows MinD during the oscillations. MinD itself only binds to the membrane in the ATP bound form [46]. MinE binds to MinD-ATP on the membrane and stimulates ATP hydrolysis by MinD leading to release of MinD-ADP from the membrane [47]. While diffusing in the cytoplasm MinD-ADP is then converted back to MinD-ATP which rebinds to the cell membrane at a new location. In this way, MinE chases the MinC-MinD complex giving rise to the regular oscillations. It has been demonstrated by computer simulations that these oscillations lead to higher concentration of MinC at the cell poles and lower concentration of MinC at mid-cell [48]–[60]. In this way, Z-ring formation is inhibited at the poles and only allowed at mid-cell position. The precise positioning at mid-cell depends on the nucleoid occlusion system [61]–[65]. The real situation is of course more complex than this simple picture. For example, MinE is not uniformly distributed, rather MinE forms a dynamic ring that wanders from pole to pole [32], [66], [67]. Furthermore, it has been shown that FtsZ forms a helical structure on the membrane that performs an oscillatory movement itself and this movement is then affected by the Min oscillation [68].

In cells without functional Min system the dynamics of FtsZ assembly is different and in FRAP experiments the recovery time of the Z-ring is longer than in wild type (WT) cells [69]. This indicates that the Min system has a quite complicated effect on FtsZ polymerization. The biggest change in *minB* ^−^ cells is that Z-ring structures can form at any chromosome-free position, in particular close to the cell poles. Cell division in this case produces mini cells which contain no chromosome and are not able to grow and divide [70]. On the other hand, *minB* ^−^ cells can also become filamentous. In total, positioning of division sites is highly irregular giving rise to a distribution of different cell sizes. Interestingly, the corresponding size distribution of a population of *minB* ^−^ cells can be explained by a simple model developed in Ref. [71]. It is based on the assumption that division at the poles effectively inhibits division at mid-cell by recruiting the division machinery away from the mid-cell positions. The good agreement between the calculated and the experimentally measured length distribution indicates that the oscillations of the Min system would not be required if there was a different way of preventing cell division close to the cell poles. Indeed, in other bacteria, such as *Bacillus subtilis*, the Min system does not perform oscillations but is statically attached to the cell poles and division septum [72], [73].

As mentioned, the Min system is the best-studied spatial oscillator. However, we show here that it also influences timing of cell division. In the absence of a functioning Min system not only the positioning of the cell division site but also the time between two sequential division events becomes irregular. To study this effect in a quantitative way, we measure the time difference between the appearance of a division site and the division event as well as the Z-ring existence time. Both quantities are found to be different in cells with and without functional Min system. To interpret these findings we develop a series of theoretical models whose predictions are compared with the experimental findings. More specially, we introduce four different models out of which two (model 3 and model 4) are able to explain the experimental data for the Min mutant. Model 4 is conceptually somewhat different from models 1-3 but is the only one that can be used to describe the WT data. We also present here the unsuccessful models 1 and 2 since from their failure important conclusions can be drawn. Our results indicate that the Min system affects the septum formation rate. In the absence of the Min proteins this rate is reduced. Together with the chromosome segregation defect this leads to the observed strongly randomized cell division events and the longer division waiting times.

## Results

**Figure 1:**
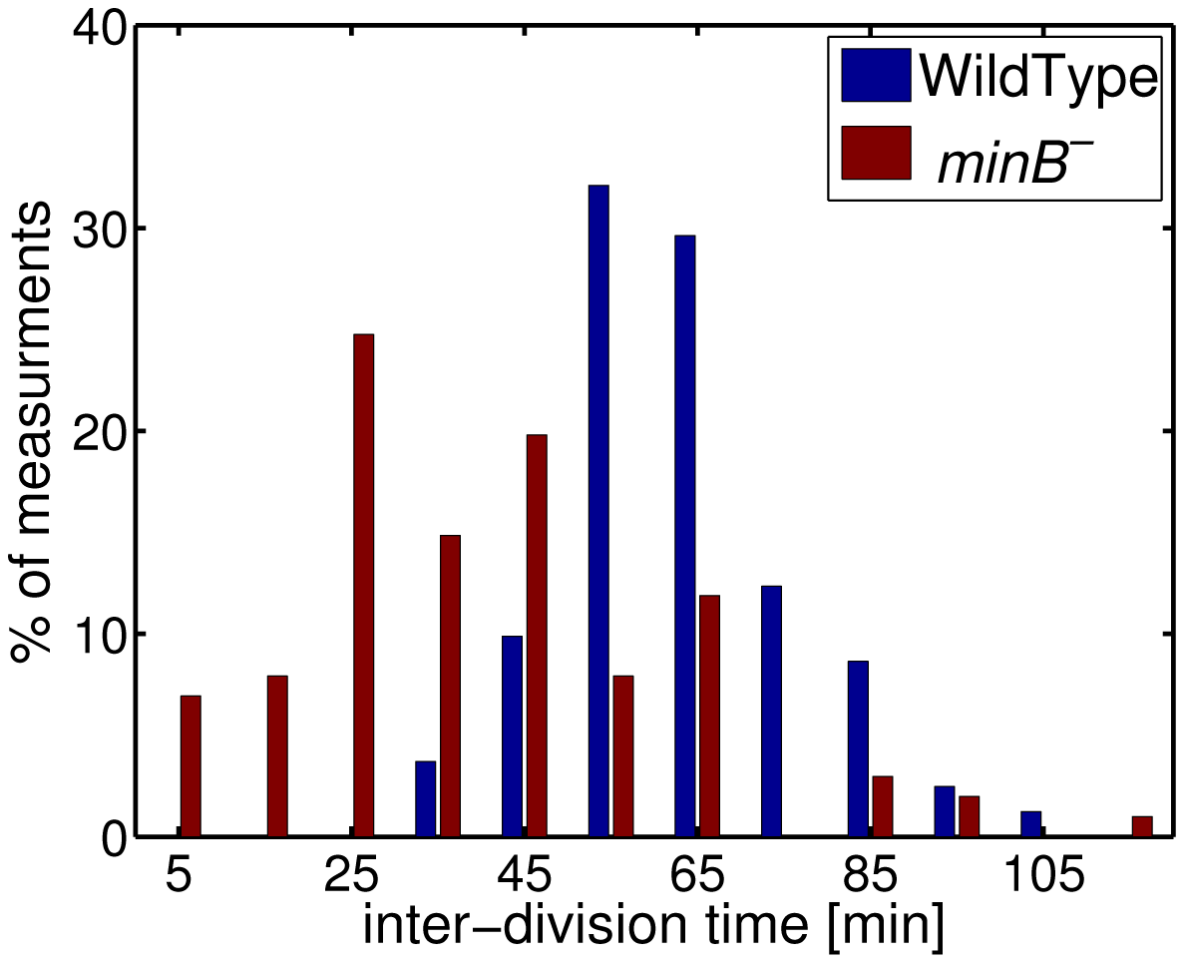
Distribution of inter-division times. Data for WT are shown in blue, for *minB* ^−^ cells in red. Histogram was obtained by observing 81 WT and 101 *minB* ^−^ division events over 210 minutes.

In this study we analyze the influence of the Min system on timing of cell division. Our investigation was triggered by our experimental observation that the distribution of inter-division times of individual wild type cells (WT, strain TB28) and Min deletion mutant cells (*minB* ^−^, strain TB43 [TB28 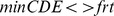]) are very different (for details about the used strains see Tables 1 and 2 and Materials and Methods). In Fig. 1 we show the distribution of inter-division times obtained from 81 WT and 101 *minB* ^−^ cells observed over 210 minutes. As can be seen the distribution is broader for *minB* ^−^ cells than for WT. To identify the origin of this we measured the time interval between chromosome segregation and cell division (in the sequel referred to as **division waiting time**) for the two strains. To track chromosome segregation, we fused the non-specific DNA-binding protein HU to GFP [74] in WT and *minB* ^−^ and treated the first visible spatial separation of two chromosomes as segregation event. Because *minB* ^−^ cells divide also at polar sites producing mini cells, we define the division waiting time of polar sites as the time interval between the formation of a cell pole and cell division at this pole.

**Table 1:** Primers.

Name	Sequence(5–3)
hupBprimerA	atcggGGATCCATCTCTAAAGCTGCGGCTGGC
hupBprimerB	atcggCCCGGGGTTTACCGCGTCTTTCAGTGCT
hupBprimerC	atcggCTGCAGgcgttgtccccagtgg
hupBprimerD	atcggGAATTCCGGCGTAGTTATTGCCTCCG
GFPfw	atcggCCCGGGgccggcggcggagccggccgatccATGGTGAGCAAGGGCGAGGA
GFPrv	atcggCTGCAGTTACTTGTACAGCTCGTCCATGCC
US-minD-up-XhoI	ATACTCGAGCGGTTTGCGGGTTATTG
US-minD-down-ClaI	CTAATCGATAGAAATTCCTTGTTAAAAAGGGA
XFP-linker-up-ClaI	CTAATCGATATGGTGAGCAAGGGCGA
XFP-linker-down-EcoRI	CGTGAATTCggatcggccggctccgccgccggcCTTGTACAGCTCGTCCATGC
minD-up-EcoRI	CGTGAATTCGCACGCATTATTGTTGTTACTT
minD-down-BamHI	ATTGGATCCAGACTTCCGGGTTGGTG
minE-up-XhoI	ATACTCGAGATGGCATTACTCGATTTCTTT
minE-down-ClaI	CTTATCGATTTTCAGCTCTTCTGCTTCC
XFP-up	atcggATCGATgccggcggcggagccggccgatccATGGTGAGCAAGGGCGAGGA
Pvenc-down-EcoRI	CGTGAATTCTTACTTGTACAGCTCGTCCA
DS-minE-up-EcoRI	ATAGAATTCGCCCGCTGTAAAAGCG
DS-minE-down-BamHI	TAAGGATCCCAAAAAAAGCCCGCC

**Table 2:** Strains and plasmids.

Strain	Description	Source
TB28	MG1655 *lacZYA* 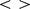 *frt*	(Bernhardt and Boer, 2003 [86])
TB43	TB28 *minCDE* 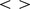 *frt*	(Bernhardt and Boer, 2005 [64])
TB28 Hu-GFP	TB28 *hupB* 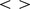 *hupB-egfp*	This work
TB43 Hu-GFP	TB43 *hupB* 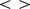 *hupB-egfp*	This work
TB28 Hu-mCherry	TB28 *hupB* 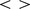 *hupB-mCherry*	This work
TB43 Hu-mCherry	TB43 *hupB* 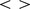 *hupB-mCherry*	This work
TB28 mCherry-MinD	TB28 *minD* 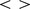 *mCherry-minD*	This work
TB28 MinE-venus	TB28 *minE* 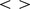 *minE-venus*	This work
TB28 mCherry-minD 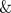 minE-venus	TB28 *minD* 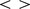 *mCherry-minD minE* 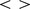 *minE-venus*	This work
DH5 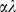 pir	80d*lacZ* M15 (*lacZYA-argF*)*U196 recA1 hsdR*17 *deoR thi-1 supE*44 *gyrA96 relA1/pir*	(Miller and Mekalanos, 1988 [87])
pJC68	P_208_-*ftsZ-eyfp*	(Jon Beckwith,2001 [85])
pBlueskript II SK-	cloning vector	Fermentas
pNPTS138-R6KT	*mobRP*4^+^ 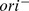 *R6K sacB*	(Lassak et al, 2010 [84])
pCHYC-2		(Thanbichler, Iniesta and Shapiro, 2007 [88])
pVENC-2		
pGFPC-2		

To avoid complications in WT cells arising from multiple partially replicated chromosomes, we grew cells in poor nutrition medium (M9 plus 1% Casamino acid (CAA) and 0.5% glycerol) at 30°C. As can be seen from the OD plots in Fig. S1 in File S1, lack of the Min system does not lead to a visible growth defect.

**Figure 2:**
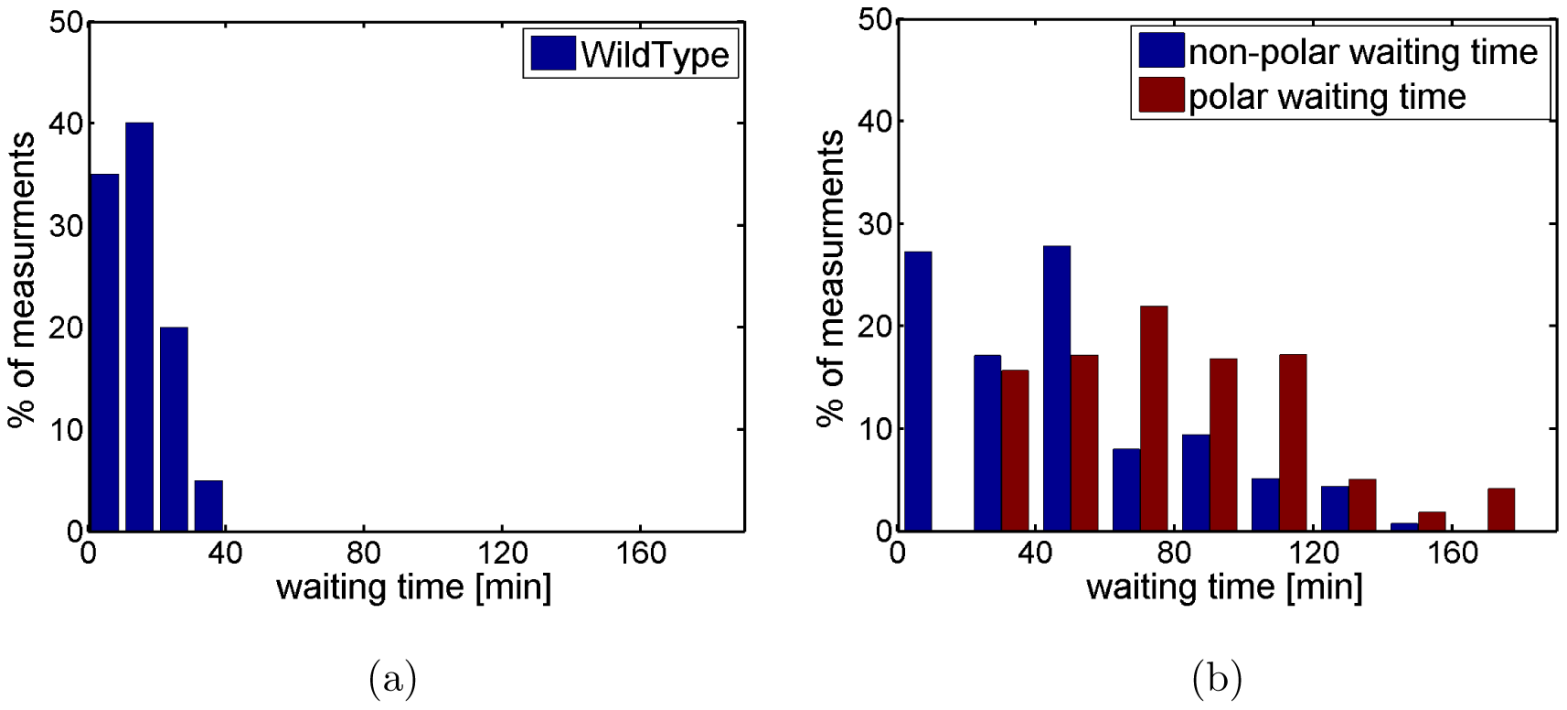
Distribution of division waiting times. Data are for WT (a) and *minB* ^−^ (b). For *minB* ^−^ one has to distinguish between polar sites (red bars) and non-polar sites (blue bars). Data were obtained by observing 60 WT and 77 polar and 100 non-polar division events in *minB* ^−^ cells over 213 minutes.

The measured division waiting times for both strains are shown in Fig. 2. As one can see, the division waiting times of *minB* ^−^ (Fig. 2b) are generally longer and show more variation than those of WT (Fig. 2a). Furthermore, for *minB* ^−^ the division waiting times of polar sites are generally longer than that of non-polar sites. Thus, the absence of the Min system not only affects positioning of division site but also timing of the division event.

To understand these findings in a quantitative way, we developed a simple model for cell growth and cell division that we applied to the *minB* ^−^ and WT cells. Our model (in the following referred to as model 1) is based on the following assumptions (for further details see File S1):

- Each cell has its individual doubling time 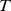 drawn from a normal distribution (75±15 min, see Sect. IIB and Fig. S9 in File S1). As we show in File S1 individual cells increase their length exponentially with time. Thus, every time step 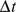 each cell increases its length by an amount (for details see Sect. IIA and Figs. S7 and S8 in File S1)

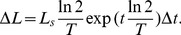
(1)Here, 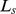 is the length of the cell at birth. Furthermore, 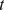 is the time since the last division event of the cell (which in the case of daughter cells corresponds to their current age). This increase in length guarantees that after time 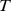 the cell length has doubled and cell mass increases exponentially with time. As shown in Fig. S2 in File S1 this leads to exponential growth of the culture with a doubling time of 75 min.
- *minB* ^−^ cells might have several chromosomes. In this case, we partition the cell into different compartments each containing a full chromosome. Thus, the cell length is given by the total length of these compartments. Each compartment is treated as an independent cell. This assumption is justified by our finding that the growth rate of individual cells depends on their length (see File S1 for details). Thus, for cells with several chromosomes the different compartments might have different doubling times. These growth rates are assigned to the compartments upon initiation of a new round of replication. Whenever two chromosomes segregate a compartment of length 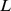 is split into two compartments of length 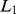 and 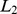, where 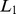 is drawn from a normal distribution (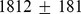 nm, see File S1) and 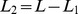. The boundary between these two compartments is a new (potential) division site. To test the validity of this assumption we performed also simulations of a modified model where all cell compartments in the culture have the same doubling time. In this case we obtained similar results with the only difference being that the simulations required somewhat more time to reach steady state.
- Cell growth and chromosome replication occur in synchrony. Thus, whenever cells (or compartments) reach their division length the chromosomes have been replicated and division waiting time is finished. For WT the division waiting time is drawn from a normal distribution with average 17.7 min and standard deviation 11.9 min (as extracted from Fig. 2a, see Sect. IIB and Fig. S10 in File S1). For *minB* ^−^ cells each division site has its individual waiting time drawn from the experimentally measured distribution (see Fig. 2b). Once a new pole appears it gets assigned a waiting time drawn from the experimental distribution (for polar sites).
- Division site placement has a random component. For WT the daughter cells have an average size of 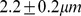 (for details see Sect. IIC and Fig. S11 in File S1). Non-polar division site placement occurs for both strains at the middle 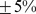 between two neighboring chromosomes. Because mini-cells are much smaller than *minB* ^−^ cells with one (or several) chromosomes we only keep track of the number of mini cells but not their size.

All of the above parameter values in the simulations are fixed by the experimental data (for details see File S1).

**Figure 3:**
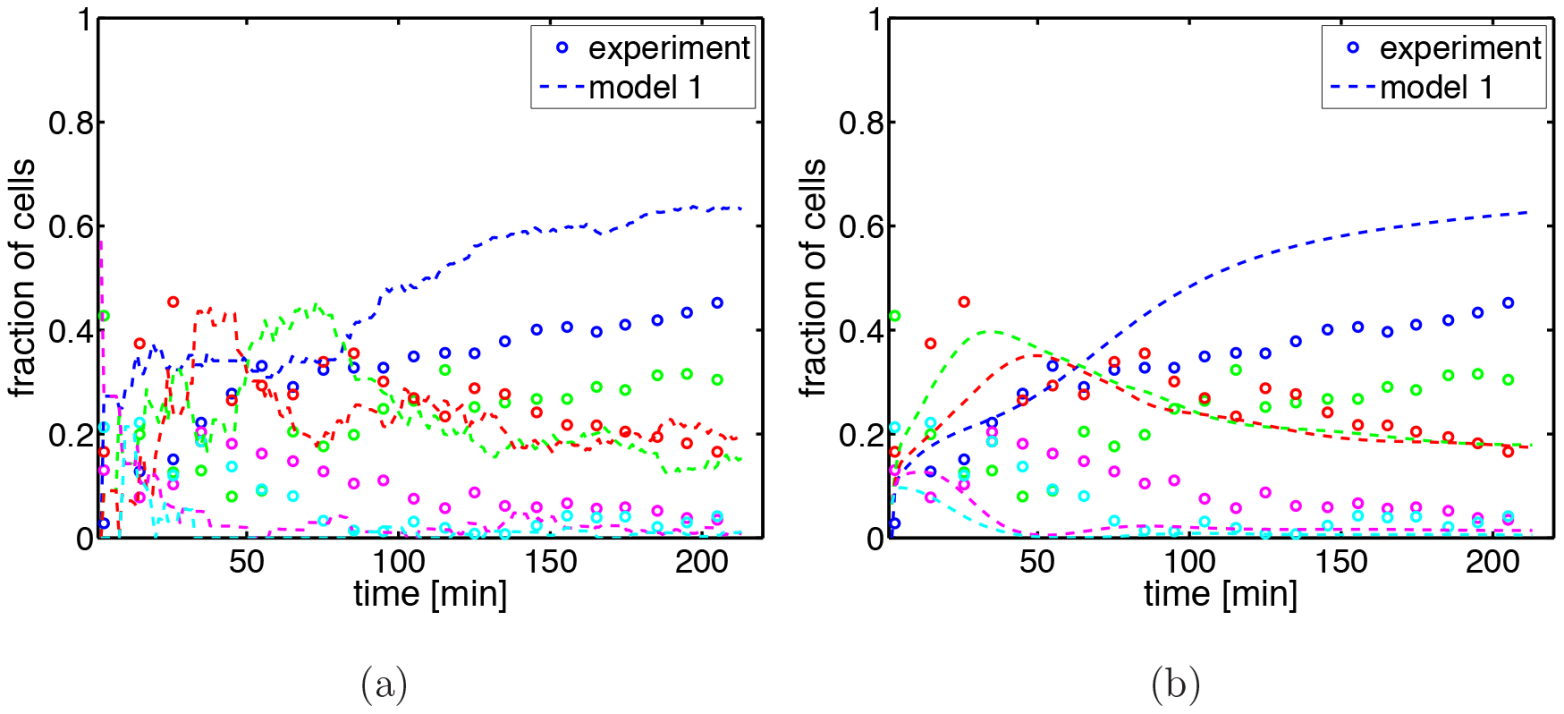
Time dependence of the fraction of cells with zero to four chromosomes. (a) In the experiments (dots) and in the simulations (dashed lines) we start with 7 cells and determine the composition of the growing population. Cells without a chromosome (mini cells) are shown in blue, cells with one chromosome in green, with two chromosomes in magenta and with four chromosomes in cyan. (b) Comparison between the experimental data and calculated data obtained from averaging over 50 simulations each one starting with 1000 cells.

To see if our model is able to capture the growth dynamics of the *minB* ^−^ cells, we performed a series of experiments in which we measured the time-dependent fraction of cells in a growing population having zero to four chromosomes (where the mini cells have no chromosomes). In these experiments we can follow the growth dynamics only for about 200 minutes since after 3–4 doubling times the agar slides, on which the cells are growing, become too crowded leading to nutrient limitation and visibly shorter cells. These measured data were compared with the simulation results of model 1. We started simulations with a number of cells that is comparable with the experimental one (about 7 cells). To our surprise we were not able to get good agreement between simulations and experiments. The best result we could achieve by adjusting the initial conditions is shown in Fig. 3a. As one can see, there are significant differences between the predicted and observed data for all fractions of the populations. We also tested if the differences could be caused by the fact that the experimental data are obtained by averaging over 2 different populations. However, even in this case the differences are larger than the standard deviations, see Fig. S3 in File S1. The differences even remain if we average over many simulations, see Fig. 3b. As one can see the dynamics shows a rather strong dependence on cell number, while the steady state values are independent of it. We therefore decided to analyze in the following only quantities that do not depend so strongly on number of cells.

**Figure 4:**
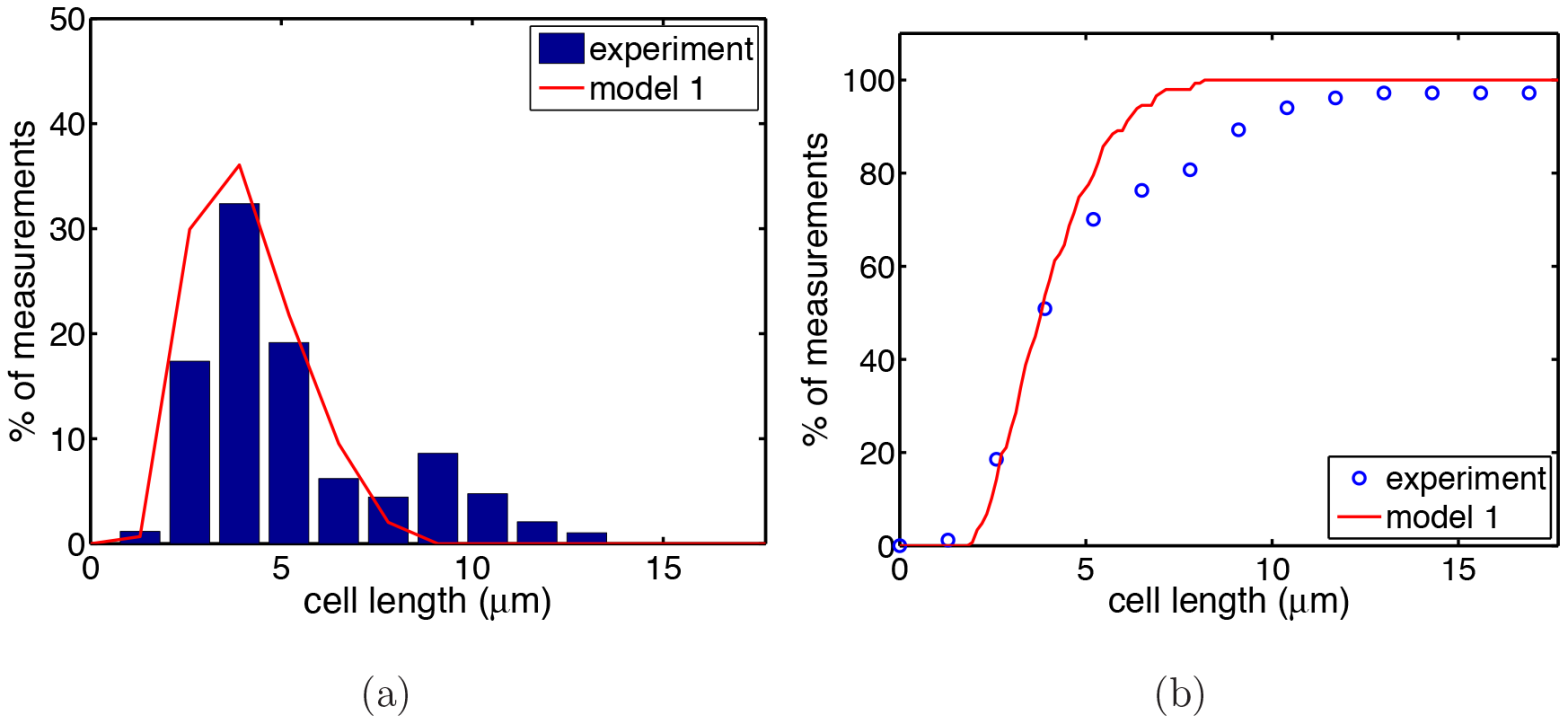
Cell length distribution of a population. Cell length distribution (a) and cumulative distribution of cell length (b) as obtained experimentally (blue bars respectively dots) and calculated from the simulations of model 1 (solid red curves). The simulations started with 7 cells. The histogram was obtained at fixed time (213 minutes after start). In the experiment 238 cells were present at that time, out of these 105 mini cells that were not taken into account.

To find the origin of the differences between model predictions and experimental data, we next tested if our model is able to reproduce the size distribution of cells. To do so we measured the distribution of cell lengths of a growing population with 7 initial cells. Fig. 4a shows the corresponding histogram. Similar results were obtained for simulations with a different number of initial cells.

As one can see, the calculated distribution (red line) fits the experiment data (blue histogram) only for small cells with sizes below 4 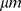. The significance of the differences becomes even more apparent by calculating the cumulative distribution of cell length (that smoothens out the effects arising from the discrete nature of the data), see Fig. 4b. This plot also shows that deviations between experiment and simulation occur for cells larger than 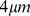. Thus, compared with the experimental system the simulation produces too few filamentous cells.

**Figure 5:**
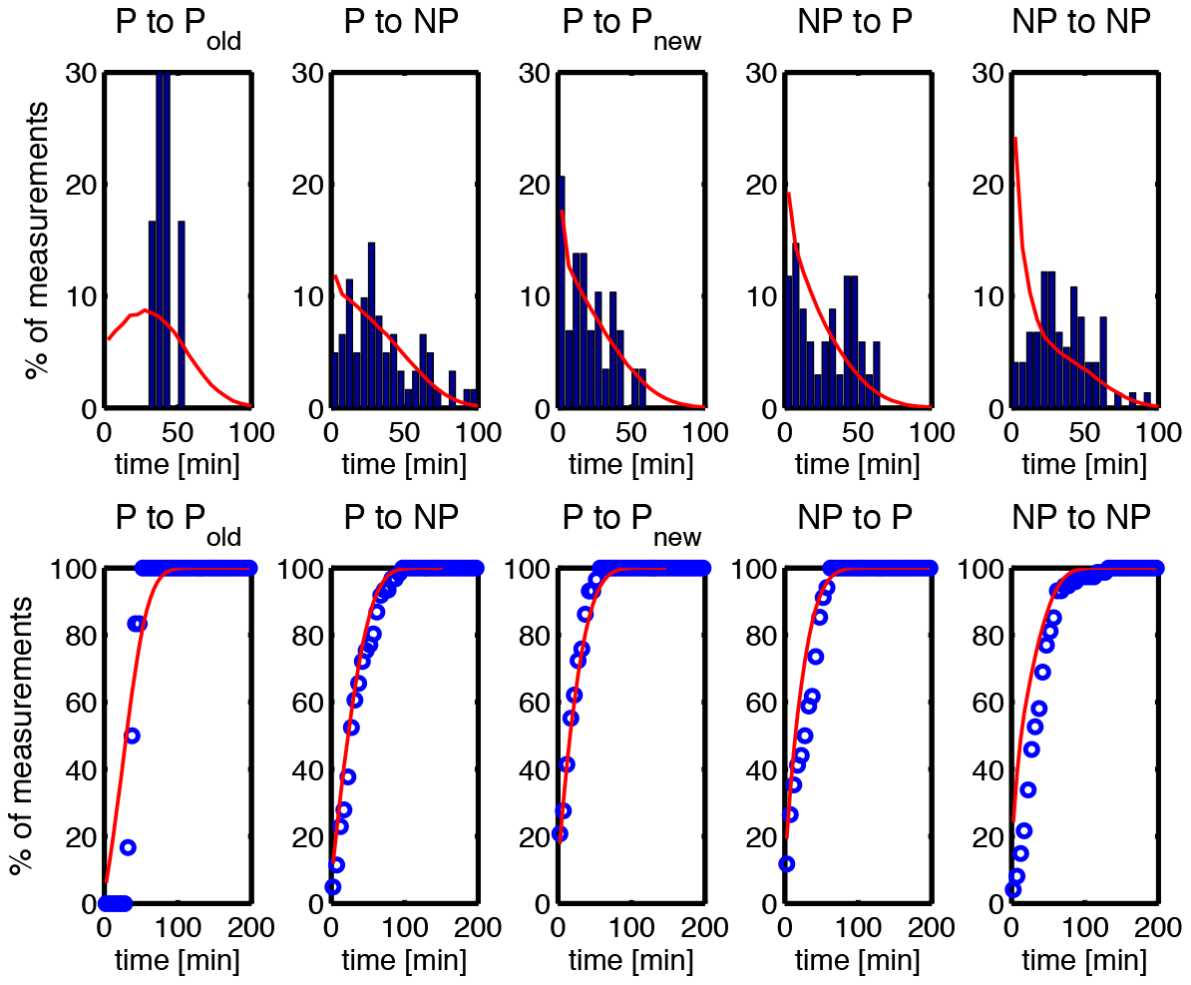
Distribution (top panel) and cumulative distribution (bottom panel) of inter-division times for different types of cell division. Cell division events are classified into 5 types according to the position of two successive cell divisions (see Table 3 for details). Blue bars represent experimental data, red lines the predictions of model 1. In the figure, P refers to polar division, NP to non-polar division. Data were obtained from 204 division events.

This indicates that there is a significant difference between model and experiment concerning cell division. To analyze if timing or positioning of cell division is the origin of this difference we analyzed the cell division history of individual cells. We measured the spatial positions of two successive division events and the time interval between these two events. To do this in a quantitative way we classified the first division event as being polar or non-polar. The second division event of the daughter cells was then classified as being either polar (and division occurs at the old or new pole) or non-polar. Simultaneously, we measured the time difference between the two division events. Table 3 summarizes our findings. As one can see, the standard deviations of the time between two division events is comparable to the mean indicating a strong cell-to cell variation. This can also be seen from Fig. 5 where we show the distribution of individual inter-division times for the five different division types.

**Table 3:** Cell division history of individual cells as obtained experimentally and from model 1.

	%	old pole	non-polar	new pole
Experiment	polar	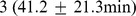	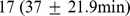	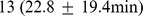
Experiment	non-polar	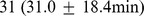	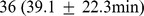	
Simulation	polar	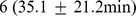	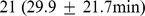	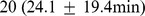
Simulation	non-polar	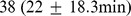	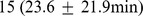	

All cell divisions within 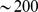 minutes are classified into 5 types according to the position of two successive cell divisions. Rows represent the location of the first division event, columns location of the second event. Number of events is given in percentage. Time in parenthesis represents mean time difference 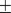 standard deviation between the division events.

These results now allow a detailed comparison between experiment and simulations. As can be seen from Table 3, in the experiments the chance of the next division occurring at a non-polar site is about 50% no matter if the previous division occurred at a polar or a non-polar site. This is different from the predictions of model 1 where the probability for a non-polar division is very low if the previous division took place at a non-polar site. This is also in agreement with the above finding that the fraction of mini cells is too high in the simulations, see Fig. 3. Furthermore, model 1 predicts a too short division waiting time after a non-polar division. This is in agreement with the above finding that in the simulations there is a too low fraction of filamentous cells.

To conclude, model 1 that is based solely on the experimentally measured waiting time distribution fails to explain the observations. In particular, the simulations yield too few filamentous cells and too many mini cells indicating that model 1 lacks essential information on where division occurs, information that is not contained in the measured waiting times which is the basis of model 1. This is also supported by the above finding that our model predicts a wrong fraction of two subsequent non-polar divisions.

**Figure 6:**
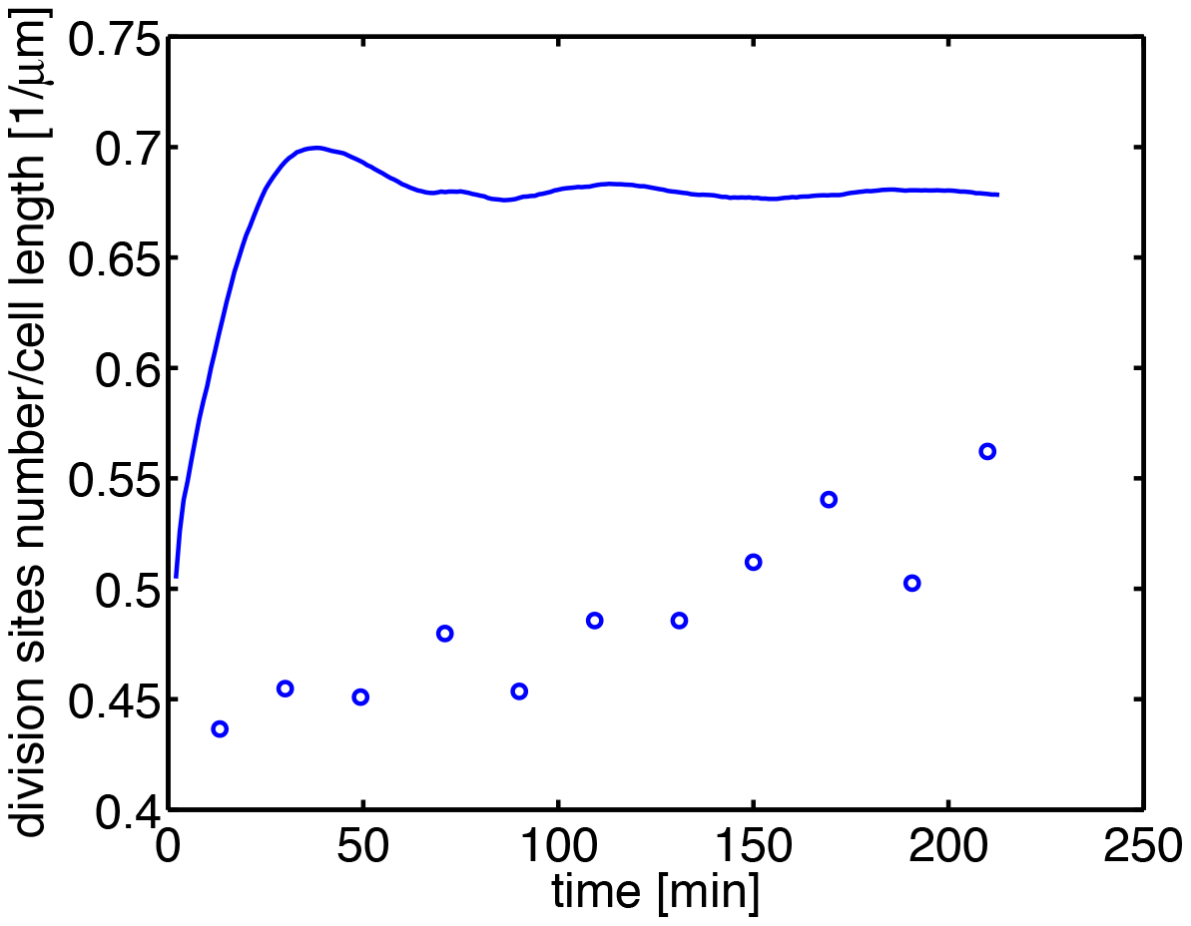
Time dependence of the number of division sites per cell length. Dots represent experimental data. 685 cells were analyzed. The solid curve is the prediction of model 1.

**Figure 7:**
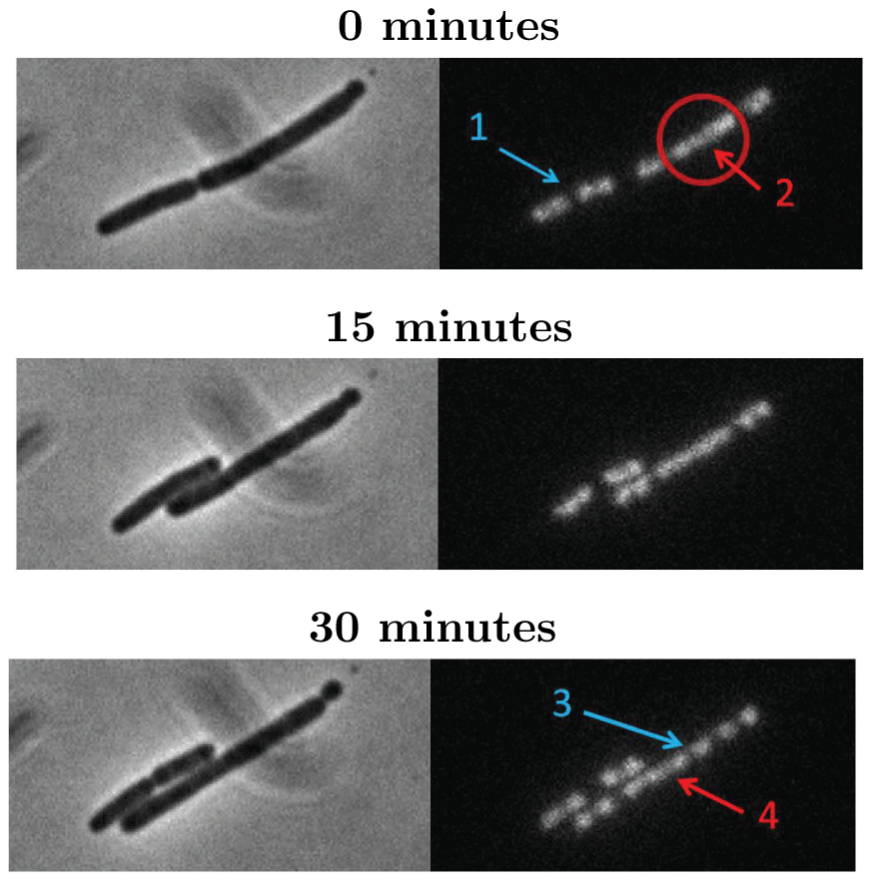
Disturbance of chromosome segregation in *minB* ^−^ cells. The phase contrast image (left column) shows a filamentous cell that divides at time 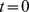. While the chromosomes are clearly separated (arrow 1) in the smaller daughter cell, the chromosomes in the larger daughter form a large cluster (arrow 2). As time proceeds the chromosomes start to segregate (arrow 3) but even after 30 minutes segregation is not complete. Some chromosomes then still form a cluster (arrow 4).

**Figure 8:**
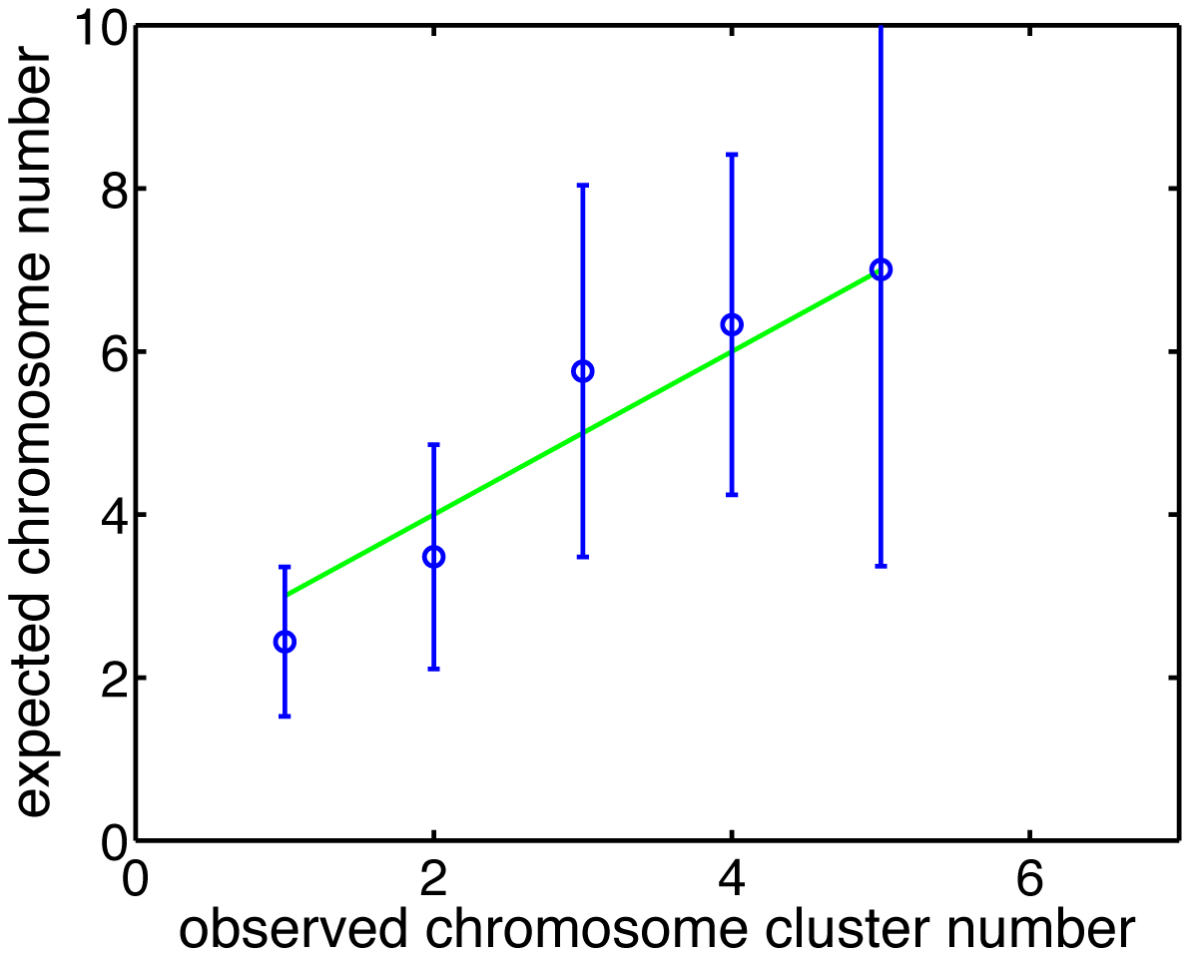
Number of chromosome clusters. The 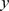-axis is the expected number of chromosome clusters 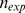 for a cell of Length 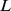. The 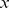-axis is the experimentally observed number of chromosome clusters 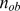. Experimental data were obtained by analyzing 685 cells observed for 220 min. The green line is the fit of the form 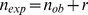, with 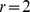.

To identify the missing component of model 1, we analyzed the number of division sites in the growing population. In doing so, we took the first clearly visible separation of two neighboring chromosomes as appearance of a division site. Fig. 6 shows the time-dependence of the number of division sites per cell length as experimentally observed and as calculated from the simulations of model 1. As one can see, there is a significant difference between the two curves, showing that in the experiments a smaller number of division sites are available. Thus, in the experiments it takes longer until a potential division site is available indicating that in the *minB* ^−^ cells chromosome segregation is less efficient. Direct observations support this finding, see Fig. 7. To quantify this effect, we calculated the number 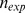 of chromosomes that we expect for a cell of Length 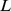 (with 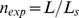, where 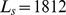
nm is the starting length) and compared this with the experimentally observed chromosome cluster number 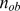, see Fig. 8. Here, a chromosome cluster corresponds either to an individual chromosome or to several non-segregated chromosomes. The data indicate that the relation between expected and observed number of chromosome clusters is best represented by 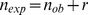, with 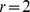. Thus, on average segregation of chromosomes does not occur at 2 of the potential division sites, i.e. 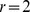 of the potential division sites are blocked. This clearly shows that the *minB* ^−^ cells have a chromosome segregation problem that is missing in model 1.

To take this effect into account we developed a new model (model 2) that extends model 1 by including the chromosome segregation defect of the *minB* ^−^ cells. Thus, model 2 also includes the experimentally observed waiting time for polar and non-polar sites. To implement the segregation defect we blocked (on average in each cell) 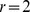 randomly picked potential division sites, see Fig. S4 in File S1.

The results of model 2 are summarized in Fig. S5 in File S1. As one can see, model 2 is in better agreement with the experimental data than model 1. However, model 2 fails to reproduce the waiting time distribution of the polar sites. This is quite surprising given the fact that model 2 is based on this distribution. However, evidently, the eventual blockage of the polar division site (because of the segregation problem) leads to too long waiting times of the polar division sites. This observation led us to speculate that the different waiting time distribution of the polar division sites is not an *a priori* property of the polar sites but rather an *emerging property*. To test this idea, we developed model 3 which is identical to model 2 except that the division waiting time of the polar sites is now drawn from the experimentally observed division waiting time distribution of the non-polar division site.

The results of model 3 are shown in Fig. S6 in File S1. As one can see, model 3 is as good as model 2 in reproducing the experimental data but additionally yields the correct waiting time distribution of the polar sites. This indicates that polar and non-polar division sites are *a priori* equivalent for cell division. However, there are additional factors that make the polar division waiting time *appear* longer. To make sure that the increase in waiting time of the polar sites is not the consequence of the fact that only specific division sites are observed, we also measured in the simulations of model 3 the waiting time distribution of division sites close to mid-cell. The waiting time of this site is nearly identical to that of the other non-polar sites indicating that there is indeed something special about the polar sites. We give possible explanations in the discussion.

**Figure 9:**
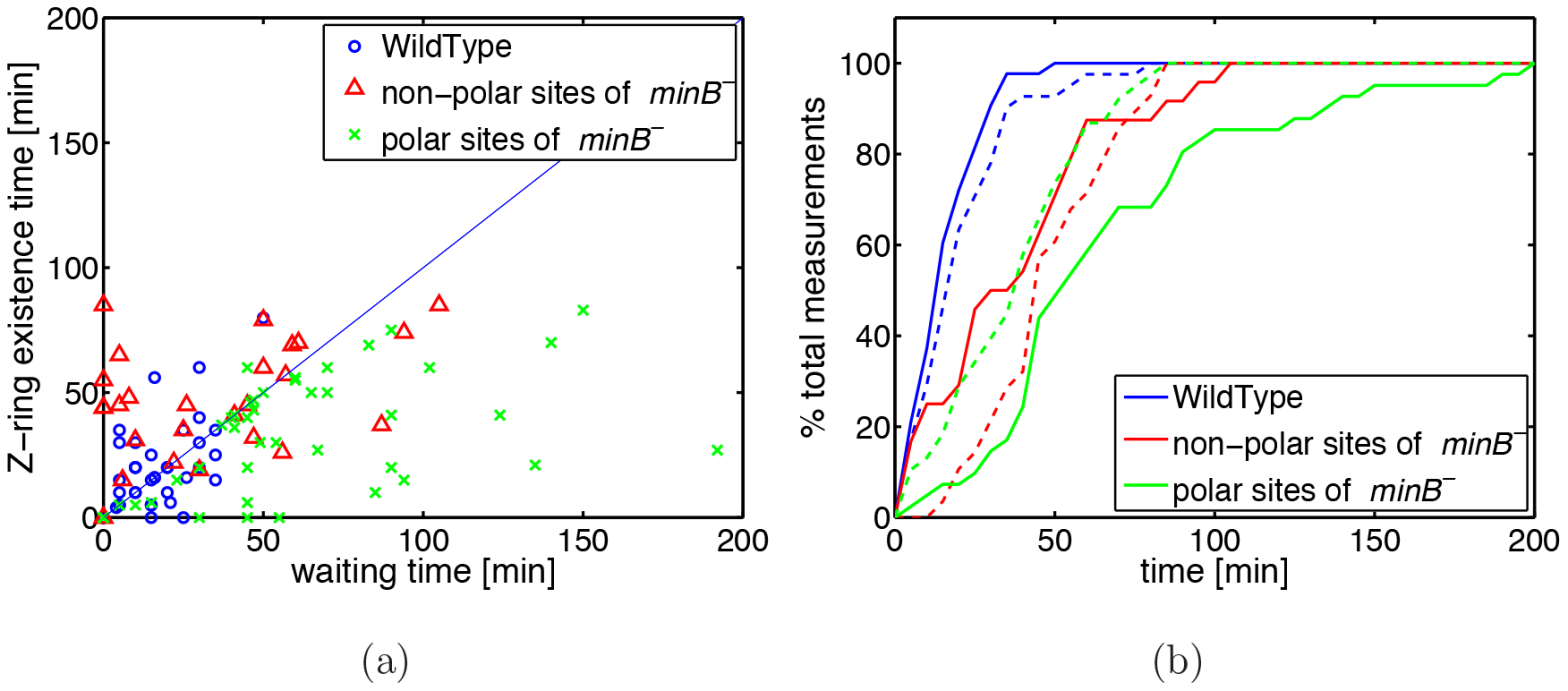
Z-ring measurements. (a) Existence time of the Z-ring as function of waiting time. Blue circles represent WT cells, green crosses the polar sites of *minB* ^−^ cells and red triangles the non-polar sites of *minB* ^−^ cells. (b) Cumulative distribution of the waiting time (solid lines) and of the Z-ring existing time (dashed lines). Same color code as in (a). Data were obtained from 41 WT cells and from 24 non-polar sites and 38 polar-sites in *minB* ^−^ cells.

The most important finding of model 3 is that there is no difference in division waiting times between polar and non-polar sites. To test this experimentally we assumed that existence time of Z-rings at a division site is a measure for the waiting time of the division site. We expressed fluorescently labeled FtsZ and determined the time interval between first appearance of the Z-ring (at a division site) and cell division at polar and non-polar sites. Fig. 9 shows this time interval as function of waiting time of the division site. As one can see, there is a clear difference between WT and *minB* ^−^ cells but no significant difference between polar and non-polar sites supporting the findings of model 3.

Thus, model 3 is able to capture the main experimental observations. But nevertheless, the question remains why *minB* ^−^ cells have a longer division waiting time than WT. We speculated that this could be caused by the fact that *minB* ^−^ cells are longer and thus have more division sites. Thus, a priory a division site in *minB* ^−^ cells has the same waiting time as a division in WT. However, because *minB* ^−^ cells have more division sites than WT it should, for a given amount of cell division machinery, take longer to finish division at these sites.

To implement this hypothesis into our model we assign a quantity 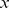 to every division site that measures how much the division process has proceeded. Upon appearance of the division site we set 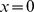, division is completed for 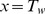, where 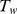 is the waiting time assigned to the division site drawn from the experimentally measured distribution of WT (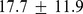
min). Between time 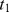 and 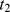 we increase 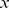 by 
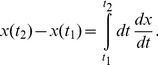
(2)


In the previous models we simply had 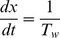 but now we want to take into account that several division sites compete for the division machinery and that larger cells have a larger amount of division machinery. We therefore set 
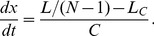
(3)


Here, 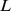 is cell length, 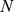 the number of potential division sites and 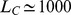
nm is the size of a chromosome. Thus, the waiting time of a site decreases the more the larger the average compartment size 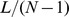 is. The constant 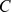 is chosen such that for WT 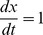, implying (see Eq. (4) below) 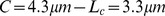. One should note that as the cell grows or as additional division sites appear 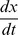 changes. Beside this novel feature model 4 is identical to model 3.

**Figure 10:**
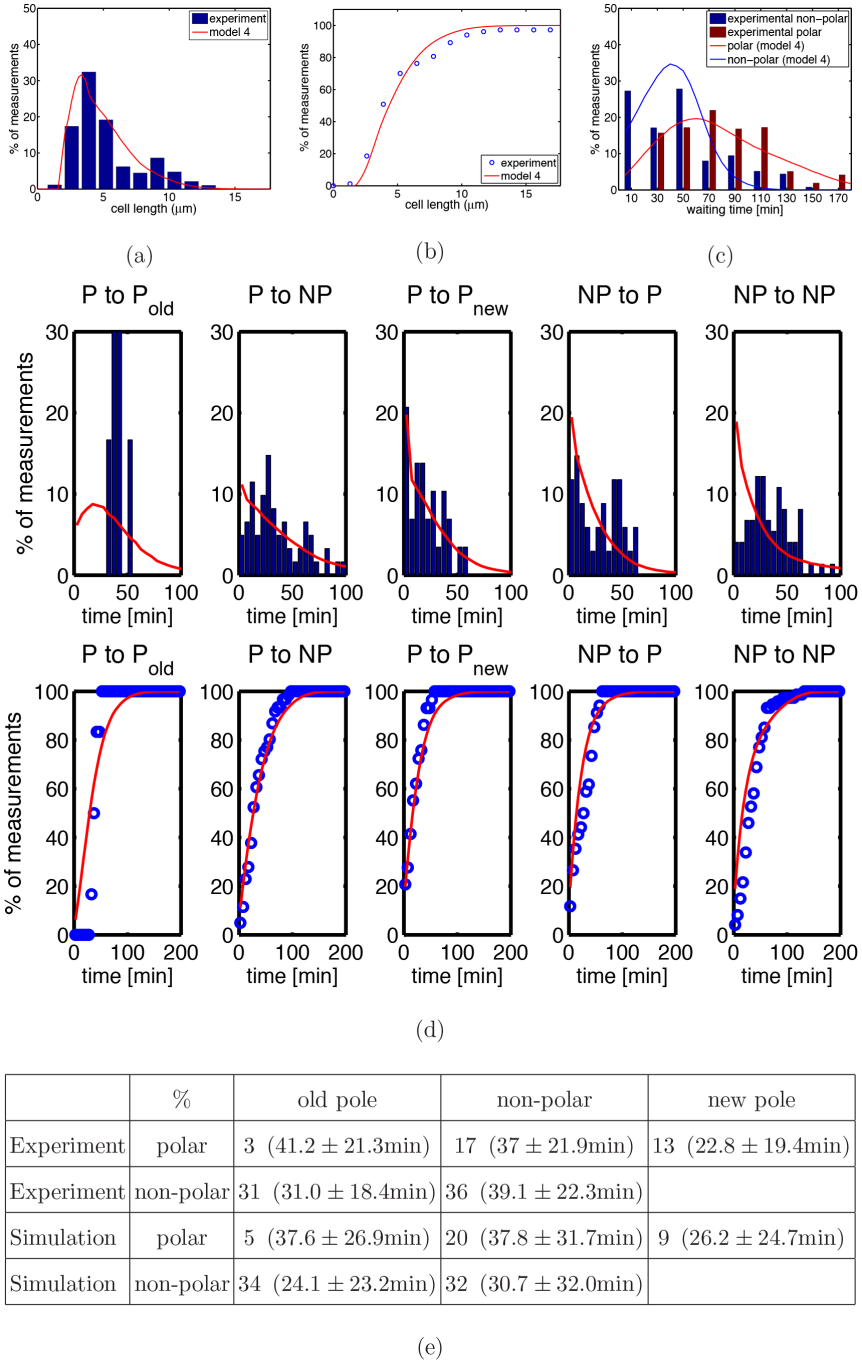
Results of model 4 for *minB* ^−^ cells. (a) Cell length distribution and (b) cumulative distribution. As in Fig. 4 bars and dots represent experimental data. Solid red lines are now the predictions of model 4. (c) Waiting time distribution of *minB* ^−^ cells for polar and non-polar sites. Solid lines are the results of model 4, bars represent experimental data (red: polar sites; blue: non-polar sites, see Fig. 4b). (d) Distribution and cumulative distribution of inter-division times for different types of cell division (same as Fig. 5 but for model 4). The cell division history of individual cells is summarized in (e), for details see Table 3.

The results of model 4 are shown in Fig. 10. As one can see, model 4 is as good as model 3 in reproducing the experimental data. Of course, the main advantage of this model is that it is independent of the experimentally measured divisional waiting time distributions.

Furthermore, we can also use model 4 to simulate WT cells. In doing so we have to take into account that the Min proteins confine the operation space of the division machinery. To implement this effect, we replace Eq. (3) by 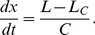
(4)


**Figure 11:**
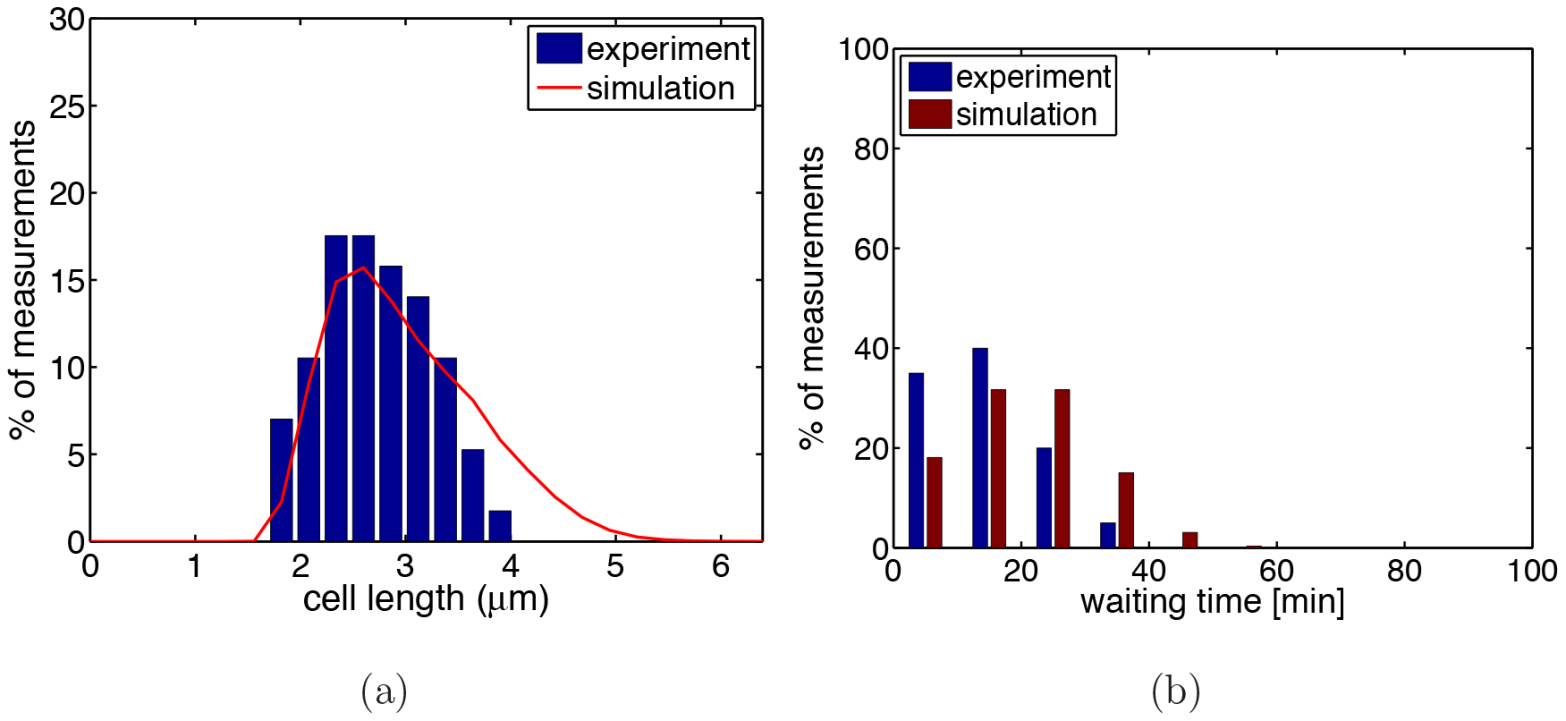
Results of model 4 for WT. Same as Fig. 10(a) and (c) but for WT instead of *minB* ^−^ cells. Experimental data in (a) and (b) were obtained from 60 and 57 cells, respectively.

In this way, the Min proteins that oscillate from pole to pole effectively confine the division machinery to a region (around the division site at mid-cell) of roughly half cell length. The predictions of model 4 for WT are shown in Fig. 11. As one can see, model 4 is also able to reproduce the experimental data for WT cells.

To perform another test of model 4 we implemented the observation made in Ref. [75] that doubling the FtsZ level in a *minB* ^−^ background gives rise to a length distribution that is quite similar to that of WT. To simulate this scenario we replaced Eq. (3) by 
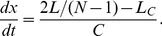
(5)


**Figure 12:**
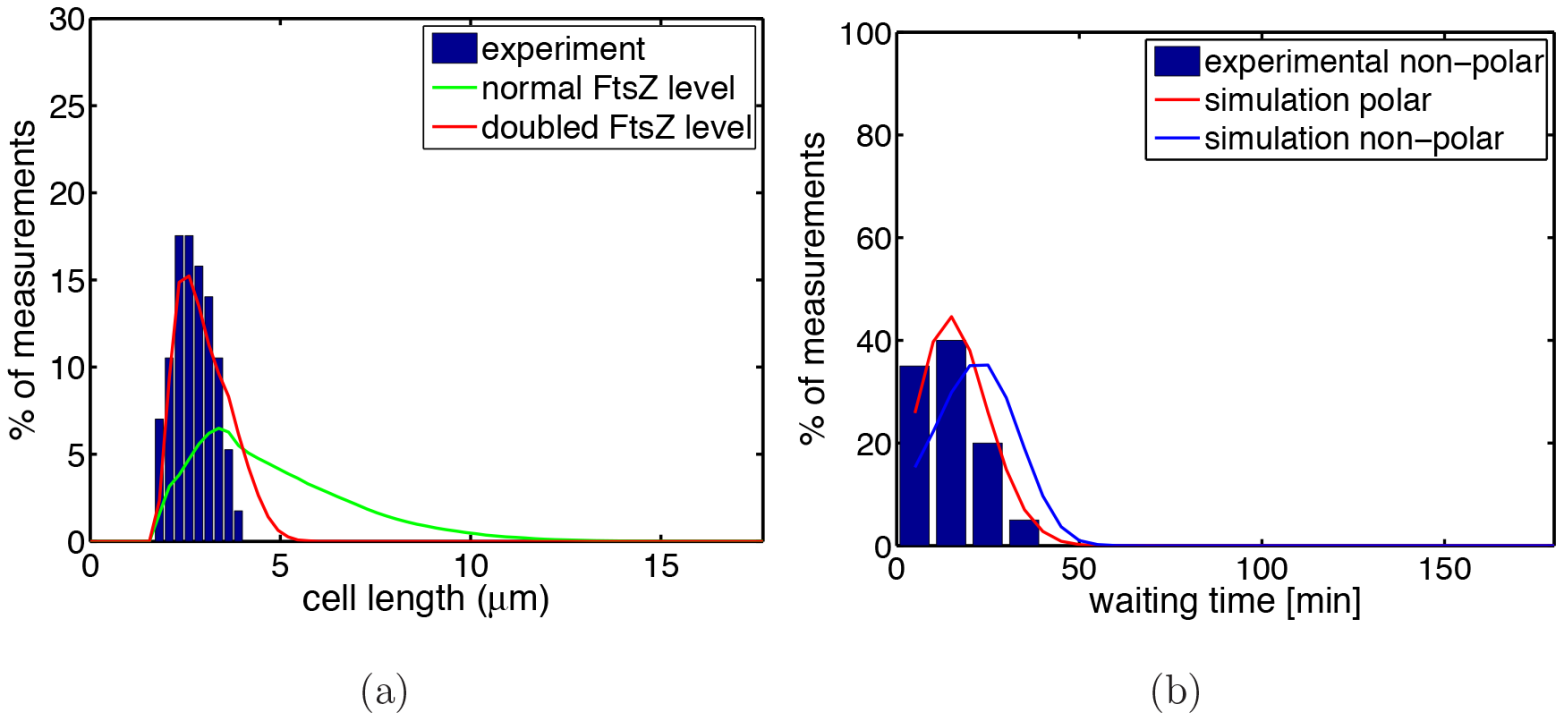
Cell length distribution of *minB* ^−^ with increased FtsZ level. As experimentally observed in Ref. [75] a two-fold increased FtsZ level in *minB* ^−^ cells gives rise to cell length distributions quite similar to that of WT. (a) Model 4 is able to reproduce this finding. In (a) blue bars represent the experimental data for WT (see Fig. 11a). Solid lines are the predictions of model 4 for normal FtsZ levels (green line) and two-fold increased FtsZ levels (red line). (b) Prediction of model 4 for the change in the waiting time distribution. Solid lines are the predictions of model 4, the blue bars the experimental data for WT (see Fig. 4a).

The factor 2 in front of 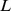 mimics the increase in FtsZ level (in this way assuming that FtsZ is the limiting factor of the cell division machinery). Furthermore, it was also shown in Ref. [75] that the distance between septa decreases upon doubling the FtsZ level. We take this as an indication that in this case the average number of blocked division sites is reduced. As one can see, in Fig. 12a model 4 is indeed able to reproduce the experimental findings. In particular, a twofold increase in FtsZ level gives also in the simulations rise to a length distribution that is quite similar to that of WT. Given this good agreement we can use model 4 to predict that also the waiting time distribution changes with FtsZ level, see Fig. 12b.

**Figure 13:**
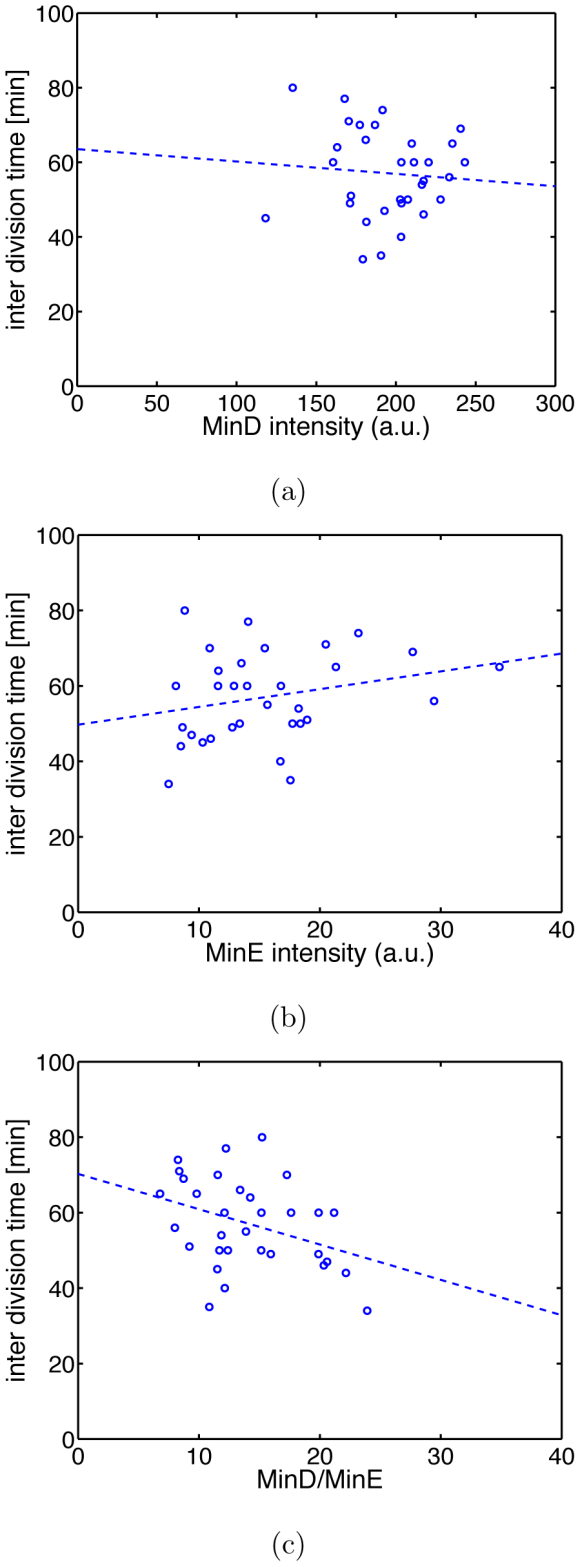
Correlation between inter-division time and MinD and MinE levels. MinD and MinE were labeled fluorescently and fluorescence intensity was taken as measure for intracellular concentration. There is no clear correlation between inter-division time and individual MinD or MinE levels [(a) and (b) with correlation coefficients 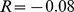 and 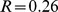, respectively], but a stronger negative correlation (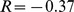) between inter-division time and the ratio of MinD to MinE level. Dashed lines are best linear fits. Data are for 32 cells.

The results obtained so far indicate that the Min systems influences timing of cell division. We were therefore wondering if the oscillation frequency of the Min system directly correlates with the inter-division times. To analyze this we fluorescently labeled MinD and MinE and measured their intensities in WT background which we take as a measure for their intracellular concentrations (for details see Materials and Methods). We found no correlation between inter-division time and the levels of MinD and only a weak influence of MinE, see Fig. 13a and b. However, there is a stronger correlation between inter-division time and the ratio of MinD to MinE, see Fig. 13c. In Ref. [51] it has been shown that this ratio determines the frequency of the Min oscillations.

## Discussion

It is well-established that the Min proteins together with the nucleoid occlusion system [61]–[63] positions the Z-ring at mid-cell [5], [31], [76], [77]. In *E. coli* the Min proteins achieve this by performing spatial oscillations. However, our results suggest that the Min proteins are not only a spatial oscillator but also have an effect on the timing of cell division.

Our interest in this aspect was triggered by the observation that in the absence of the Min system the inter-division times vary strongly between different cells. Although the corresponding distribution is strikingly different from that of WT cells (see Fig. 1) it is not so easy to quantify the irregularity of cell division timing in *minB* ^−^ cells in a meaningful way. The problem is that inter-division time itself is not an appropriate observable for this purpose as it is closely connected with the cell size which is irregular because of the lack of Min oscillations. A longer cell, that has several available division sites has a higher probability to divide earlier than a smaller cell. In order to avoid this difficulty, we studied the timing of cell division by comparing division sites rather than comparing cells. There are two major processes in cell growth that affect the timing of cell division: replication of the chromosome and septum formation between two chromosome clusters. The growth curves of WT and *minB* ^−^ strains in different liquid media at 37°C show that the Min system does not change the growth rate of the cell. This indicates that the chromosome duplication rate should be the same in *minB* ^−^ and WT strain. So to compare the timing at each division site, we only need to measure how long it takes cells to divide at each site after chromosome segregation. We refer to this quantity as division waiting time. For the cell poles the division waiting time is the time difference between the appearance of the pole and the polar division event.

Using this quantity our results show that there is a significant difference in the timing of cell division between WT and *minB* ^−^ strain, with the average division waiting time of the *minB* ^−^ cells being longer. Given that the only genotypic difference between these two strains is the lack of a Min protein, this indicates that the Min system has an effect on the timing of cell division. Furthermore, we found that the waiting time at cell poles is longer than that at other division sites (the non-polar sites).

In order to understand these findings in a quantitative way, we developed a series of four models to explain and reproduce the experimental observations. In all models we simulated the growth of a bacterial population by following the growth of individual cells. Each cell consists of one or several compartments that each contains one chromosome. Given the spatial resolution of the experimental data there is no need to incorporate into the models any details about the chromosome location and the replication process. We only keep track of the chromosomes to describe the appearance of new compartments and division sites.

Main difference between models 1–3 is the waiting distribution assigned to the division sites. In model 1 we implemented the obvious choice by simply assigning the experimentally observed distributions (see Fig. 4) to the division sites which are different for polar and non-polar sites. In model 2 this is combined with the blockage of the division sites (to mimik the segregation defect). Both models fail in explaining all relevant experimental data. Model 1 does not yield the correct cell length distribution (see Fig. 4), while model 2 fails in reproducing the correct division waiting time distribution of the polar sites, see Fig. S5 in File S1. Only model 3, where the polar division sites get the same division waiting time assigned as the non-polar sites, yields the correct distribution.

From the failure of model 1 and 2 (and the success of model 3) two conclusions can be drawn.

- The chromosome segregation defect caused by the absence of a functional Min system effectively reduces the number of available non-polar division sites.
- Polar and non-polar division sites have the same *a priori* probability of being picked as a division site.

The chromosome segregation defect in *minB* ^−^ cells has also been observed in other studies [70], [78], [79]. The mechanism of this effect is not clear yet, but evidently Min seems to play an important role in chromosome segregation [80], [81]. In the models we use a rather simple description of the segregation defect as an effective blockage of (on average) two potential division sites. However, the agreement between model 3 and the experimental data shows that this description is appropriate for our purposes. For a more sophisticated description more details about the segregation defect need to be known.

Finding (ii) is the consequence of the failure of model 2. From the analysis of this model it becomes clear that one has to distinguish between the assigned waiting time distribution (i.e. the waiting time that is assigned to every division site) and the apparent or emerging division site distribution that is obtained by measuring the waiting time of every division site.

Thus, *a priory* polar and non-polar division sites are equivalent but apparently, the chromosome segregation defect has different consequences for polar and non-polar sites. For the non-polar sites chromosome segregation is clearly visible. However, it is less clear when a division site appears at the poles since it is difficult to distinguish between chromosome fluctuations and segregation.

Our finding that timing of polar and non-polar division is identical is further supported by our measurements of the lifetime of the Z-ring. As we saw the lifetime distribution of a Z-ring is comparable at polar and non-polar sites in *minB* ^−^ cells. As for the waiting time the lifetime of a Z-ring, i.e. the time interval between initiation of Z-ring formation and cell division, is longer in *minB* ^−^ than in WT cells for all division sites.

These results are further support for our claim that the lack of the Min system has an effect on the timing of cell division. One can think of many different scenarios that could cause the longer division waiting time in *minB* ^−^ cells. The one we prefer here is that the formation rate of the septum is reduced. It is based on the idea that in the absence of Min oscillations there are more available positions for FtsZ to assemble a Z-ring structure. At the same time the total amount of division machinery does not change, so the septum formation rate is affected. We have tested this idea by introducing model 4 where the division waiting time is set by competition of the division sites for the divisional machinery. This assumption is also supported by our experimental observations that in *minB* ^−^ cells sometimes Z-ring structures were quite extended possibly consisting of several ring structures. Because of this competition the division waiting time in model 4 depends on cell length and compartment number. Here, the cell length represents the amount of division machineries in each cell, while the compartment number relates to the available space for Z-ring assembly.

Thus, model 4 is conceptually somewhat different from models 1–3 since it is not based on the experimentally observed waiting time distributions of *minB* ^−^. Rather, the waiting times are directly calculated from the process of septum formation and the WT distribution of division waiting times. This allows us to use model 4 to describe the properties of WT cells as well. To do so we only have to include that FtsZ is prevented from forming the Z-ring at the poles. Interestingly, even in WT the Min system has an effect on timing of cell division. As shown in Fig. 13c, the inter-division time of WT cells depends on the ratio of concentrations of MinD to MinE that is believed to set the frequency of the Min oscillations [51]. Thus, the emerging picture is that the Min system has a crucial influence on the septum formation rate and that in the absence of the Min proteins this rate is reduced.

This hypothesis is also supported by the finding that *minB* ^−^ cells with a doubled amount of FtsZ have a length distribution quite similar to that of WT. This was experimentally observed in Ref. [75]. Model 4 yields similar results, see Fig. 12. Our model also predicts that for doubled FtsZ level the waiting time distribution of the division sites of *minB* ^−^ cells becomes similar to that of WT cells. All these findings indicate that the change of the rate of septum formation probably mainly relates to differences in the efficiency of Z-ring formation.

A molecular understanding of the influence of the Min system on septum formation is beyond the scope of this paper. It is clear from earlier studies that assembly of FtsZ on the cell membrane is affected by the Min system in a complex manner. The stiffness of FtsZ structures is affected by MinC, and so is the function of the Z-ring as a scaffold [42]. Furthermore, both experimental and theoretical approaches have shown that FtsZ can form helical structures [68], [82]. These structures are then pushed by MinC to oscillate in the cell as well [68]. But without the Min system, the assembly of FtsZ is different. In the absence of Min the recovery time of the Z-ring after photo-bleaching is twice as long as in WT indicating that the Z-ring is more dynamical in WT cells [69]. In a more molecular model all these details need to be taken into account.

To conclude, our results show that the Min proteins have an important influence on timing of cell division. This indicates that the Min system is not only a spatial oscillator but also has some aspects of a temporal oscillator (which, according to the definition given above, determines when cellular events take place). Of course, there are important differences to the most prominent examples of temporal oscillators (the cell cycle oscillator or the circadian oscillator). There, temporal oscillations are implemented by temporally varying the concentration of active proteins throughout the cell. This requires some molecular mechanism to control protein function, either through production and degradation of protein or through regulation of protein activity. For the Min system this is not the case, but because of the spatial oscillations the concentration of Min proteins at the (potential) division sites varies with time. If the Min system is indeed a temporal oscillator then one expects that the inter-division time (that is being set by the oscillator) depends on the frequency of the Min oscillations. Our results already indicate such a dependency indirectly as the inter-division time depends on the ratio of MinD to MinE that is believed to determine the frequency [51]. However, this point remains to be proven experimentally.

## Materials and Methods

### Cell growth

For cloning purposes, *E. coli* cells were grown at 37°C in LB liquid medium or on LB plates containing 1.5% agar. When appropriate, antibiotics were added at the following concentrations (g/ml; liquid/solid): ampicillin (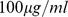), kanamycin (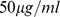). Liquid cultures were incubated shaking at 220 rpm at 37°C. Cultures for microscopy experiments were grown in M9 minimal medium [83] with 0.5% glycerol instead of glucose and 1% Casamino acid, incubated shaking at 170 rpm and 30°C. The solid media included 1% agar. In order to induce expression of the *ftsZ-yfp* fusion harbored by pJC68, 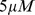 of IPTG together with 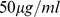 ampicillin were added to the liquid or solid media [58].

### Microscopy and image analysis

For microscopic analysis, cells were transferred onto pads made of M9 minimal medium [83] with 0.5% glycerol instead of glucose and 1% Casamino acid and 1% agar. Images were taken with a Leica DM6000B microscope equipped with a Leica Plan Apo 100/NA 1.40 phase-contrast oil objective and a Cascade II: 1024 camera (Photometrics). During the course of the analyses, the temperature of the slide was kept constant with a circulating water jacket objective heater, whose temperature was controlled by a water bath (ministat CC3, Huber) and a feedback system. Images were recorded with Image Pro 6.2 (Media Cybernetics) and processed with Metamorph 7.7.5.0 software (Molecular Devices).

Data analysis was performed with ImageJ v1.42q. Cell lengths and chromosome cluster lengths were measured with the straight/segment line selections tool and the cell outlines were manually marked with the freehand selection tool. The fluorescence intensities of the mCherry, Venus and YFP signals were obtained by integrating over this area.

### Strains and plasmids

To generate derivatives of strains TB28 and TB43 carrying a fusion of the endogenous *hupB* gene to *gfp* or *mCherry*, three PCR fragments were amplified: the AB fragment (the C-terminal part of *hupB*) was amplified from *E. coli* chromosomal DNA with the primers hupBprimerA and hupBprimerB, the CD fragment (the downstream region of hupB) was amplified from *E. coli* chromosomal DNA with the primers hupBprimerC and hupBprimerD and the *egfp/mCherry* sequence was amplified with the primers GFPfw and GFPrv from the plasmids pGFPC-2 or pCHYC-2. In several cloning steps, the AB fragment was fused with the respective fluorescent protein gene and the CD fragment and ligated into plasmid pBluescript II SK-, using BamHI, SmaI, PstI and EcoRI restriction sites that were introduced during the PCR reactions (see primers in Table. 2). A fragment containing the three fragments was released from the resulting plasmid by restriction with BamH-I and EcoR-I and ligated with the equally cut plasmid pNPTS138-R6KT. The resulting plasmid was then transformed into strains TB28 and TB43, and the fluorescent protein sequence was finally fused to *hupB* on the chromosome by double homologous recombination using a two-step protocol based on sucrose counterselection [84].

To construct a derivative of TB28 that synthesizes MinD labeled with mCherry (red fluorescent protein) and MinE labeled with Venus (yellow fluorescent protein), we first PCR-amplified the upstream region of *minD* and the 5′ region of *minD* from *E. coli* chromosomal DNA using the primers pairs US-minD-up-XhoI/US-minD-down-ClaI and minD-up-EcoRI/minD-down-BamHI. Then the *mCherry* sequence from the plasmid pCHYC-2 was amplified with the primers XFP-linker-up-ClaI and XFP-linker-down-EcoRI. These three fragments were then sequentially ligated into the plasmid pBlueskript II SK- using terminal restriction sites that had been introduced during the PCR amplification step (see primers in Table 2). Then, a fragment containing the three fragments was generated by digesting the resulting plasmid with Apa-I and BamH-I and inserted into equally treated plasmid pNPTS138-R6KT. The plasmid was then transformed into TB28, and the fluorescent protein sequence was finally fused to the 5′ end of *minD* on the chromosome by double homologous recombination. In a second step, the primers minE-up-XhoI, minE-down-ClaI, XFP-up, Pvenc-down-EcoRI, DS-minE-up-EcoRI and DS-minE-down-BamHI were used to amplify the 3′ region of *minE*, the gene encoding the fluorescent protein Venus, and the downstream region of *minE* from *E. coli* chromosomal DNA. The three fragments were sequentially ligated into plasmid pBlueskript II SK-. Subsequently, the fused fragments were transferred into pNPTS138-R6KT using the procedure described above, and the Venus sequence was fused to the 3′ end of *minE* on the *E. coli* chromosome.

The construction of a strain synthesizing an FtsZ-YFP fusion was performed as described in Ref. [85]. The corresponding plasmid was a gift of B. di Ventura.

All primers and strains used in this study are summarized in Tables 1 and 2, respectively.

### Simulations

If not states otherwise the simulations of the models started with 1000 randomly initialized cells with different numbers of chromosomes and cell lengths at time 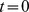. Each cell contains one or several compartments accordingly set by its number of chromosomes. To determine when the chromosome is ready to segregate, we assign each new chromosome a time 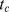 that is reduced every time step. At 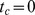 the chromosomes is ready to segregate. The time 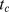 is given by 
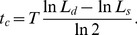
(6)


Here, 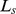 and 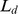 are, respectively, the starting and ending length of the corresponding compartment, and 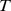 is the doubling time. A simulation step represents one time step. In every time step 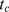 is reduced by one for all compartments and cell length is increased by 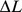 as given by Eq. (1). At every time step all chromosomes with 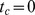 are duplicated completely. In model 1, all chromosomes segregate giving rise to new potential division sites. In model 2–4, random numbers 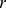 between 0 and 1 are distributed to the boundaries between neighboring chromosomes. The sites where 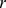 is larger than some threshold value 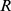 are blocked and there the chromosomes do not segregate. At all other sites the chromosomes segregate giving rise to new potential division sites. To guarantee that (on average) only two of the potential division sites are blocked we set for a cell that has 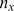 potential division sites blocked 
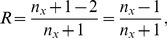
(7)whenever a new site appears. New random numbers are then assigned to the blocked and the new potential division site.

Initially, we assign all non-blocked division sites a waiting time 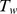. In models 1–3, 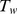 is drawn from different normal distributions for polar and non-polar sites (with 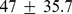
min for non-polar sites and 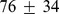
min for polar sites). In model 4, 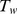 is drawn from the waiting time distribution of WT cells (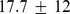
min). A simulation step represents one time step and in every time step 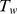 is reduced by 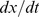, as explained in the main text. Whenever 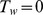 for a site then the cells divides there.

To mimic the presence of noise in division site placement, 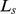 is drawn from a normal distribution (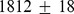
nm). Thus, one daughter cell has starting length 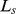 the other 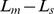 (where 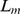 is the length of the mother cell) provided that 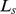 only deviates at most 10% from 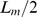.

For the simulations custom written C-programs were used. Figures were made with Matlab.

## Supporting Information

File S1 **Combined file including theoretical background information and supporting figures.**
(PDF)

## References

[1] Murray AW (2004) Recycling the cell cycle: Cyclins revisited. Cell 116: 221–234. 14744433 10.1016/s0092-8674(03)01080-8

[2] Laub MT, McAdams HH, Feldblyum T, Fraser CM, Shapiro L (2000) Global analysis of the generic network controlling a bacterial cell cycle. Science 290: 2144–2148. 11118148 10.1126/science.290.5499.2144

[3] Biondi EG, Reisinger SJ, Skerker JM, Arif M, Perchuk BS, et al (2006) Regulation of the bacterial cell cycle by an integrated genetic circuit. Nature 444: 899–904. 17136100 10.1038/nature05321

[4] Iniesta AA, McGrath PT, Reisenauer A, McAdams HH, Shapiro L (2006) A phospho-signaling pathway controls the localization and activity of a protease complex critical for bacterial cell cycle progression. Proc Natl Acad Sci USA 103: 10935–10940. 16829582 10.1073/pnas.0604554103 PMC1544152

[5] Lenz P, Søgaard-Andersen L (2011) Temporal and spatial oscillations in bacteria. Nature Rev Microbiol 9: 565–577. 21760621 10.1038/nrmicro2612

[6] Nakajima M, Imai K, Ito H, Nishiwaki T, Murayama Y, et al (2005) Reconstitution of circadian oscillation of cyanobacterial KaiC phosphorylation in vitro. Science 308: 414–415. 15831759 10.1126/science.1108451

[7] Qin X, Byrne M, Xu Y, Mori T, Johnson CH (2010) Coupling of a core post-translational pacemaker to a slave transcription/translation feedback loop in a circadian system. PLoS Biol 8: e1000394. 20563306 10.1371/journal.pbio.1000394 PMC2885980

[8] Laub MT, Shapiro L, McAdams HH (2007) Systems biology of *Caulobacter* . Annu Rev Genet 41: 429–441. 18076330 10.1146/annurev.genet.41.110306.130346

[9] Jenal U (2009) The role of proteolysis in the *Caulobacter crescentus* cell cycle and development. Res Microbiol 160: 687–695. 19781638 10.1016/j.resmic.2009.09.006

[10] McAdams HH, Shapiro L (2009) System-level design of bacterial cell cycle control. FEBS Lett 583: 3984–3991. 19766635 10.1016/j.febslet.2009.09.030 PMC2795017

[11] Boye E, Nordström K (2003) Coupling the cell cycle to cell growth. EMBO Reports 4: 757–760. 12897798 10.1038/sj.embor.embor895 PMC1326335

[12] Koch AL (2002) Control of the bacterial cell cycle by cytoplasmic growth. Critical Reviews in Microbiology 28: 61–77. 12003041 10.1080/1040-840291046696

[13] Vinella D, D'Ari R (1995) Overview of controls in the *Escherichia coli* cell cycle. BioEssays 17: 527–536. 7575494 10.1002/bies.950170609

[14] Cooper S, Helmstetter CE (1968) Chromosome replication and the division cycle of *Escherichia coli* B/r. Journal of Molecular Biology 31: 519–540. 4866337 10.1016/0022-2836(68)90425-7

[15] Helmstetter CE, Cooper S (1968) DNA synthesis during the division cycle of rapidly growing *Escherichia coli* B/r. Journal of Molecular Biology 31: 507–518. 4866336 10.1016/0022-2836(68)90424-5

[16] Donachie WD (1968) Relationship between cell size and time of initiation of DNA replication. Nature 219: 1077–1079. 4876941 10.1038/2191077a0

[17] Browning ST, Castellanos M, Shuler ML (2004) Robust control of initiation of prokaryotic chromosome replication: essential considerations for a minimal cell. Biotechnol Bioeng 88: 575–84. 15470709 10.1002/bit.20223

[18] Løbner-Olesen A, Skarstad K, Hansen FG, Meyenburg Kv, Boye E (1989) The DnaA protein determines the initiation mass of *Escherichia coli* K-12. Cell 57: 881–889. 2541928 10.1016/0092-8674(89)90802-7

[19] Mahaffy JM, Zyskind JW (1989) A model for the initiation of replication in *Escherichia coli* . J Theor Biol 140: 453–477. 2559257 10.1016/s0022-5193(89)80109-2

[20] Donachie WD, Blakely GW (2003) Coupling the initiation of chromosome replication to cell size in *Escherichia coli* . Current Opinion in Microbiology 6: 146–150. 12732304 10.1016/s1369-5274(03)00026-2

[21] Haeusser DP, Levin PA (2008) The great divide: coordinating cell cycle events during bacterial growth and division. Current Opinion in Microbiology 11: 94–99. 18396093 10.1016/j.mib.2008.02.008 PMC2397022

[22] Nordström K, Bernander R, Dasgupta S (1991) The *Escherichia coli* cell cycle: one cycle or multiple independent processes that are co-ordinated? Molecular Microbiology 5: 769–774. 1857202 10.1111/j.1365-2958.1991.tb00747.x

[23] Creutziger M, Schmidt M, Lenz P (2012) Theoretical models for the regulation of dna replication in fast-growing bacteria. New Journal of Physics 14: 095016.

[24] Leonardy S, Miertzschke M, Bulyha I, Sperling E, Wittinghofer A, et al (2010) Regulation of dynamic polarity switching in bacteria by a Ras-like G-protein and its cognate GAP. EMBO J 29: 2276–2289. 20543819 10.1038/emboj.2010.114 PMC2910265

[25] Zhang Y, Franco M, Ducret A, Mignot T (2010) A bacterial ras-like small GTP-binding protein and its cognate GAP establish a dynamic spatial polarity axis to control directed motility. PLoS Biol 8: e1000430. 20652021 10.1371/journal.pbio.1000430 PMC2907295

[26] Ebersbach G, Ringgaard S, Møller-Jensen J, Wang Q, Sherratt DJ, et al (2006) Regular cellular distribution of plasmids by oscillating and filament-forming ParA ATPase of plasmid PB171. Mol Microbiol 61: 1428–1442. 16899080 10.1111/j.1365-2958.2006.05322.x

[27] Ringgaard S, van Zon J, Howard M, Gerdes K (2009) Movement and equipositioning of plasmids by ParA filament disassembly. Proc Natl Acad Sci USA 106: 19369–19374. 19906997 10.1073/pnas.0908347106 PMC2775997

[28] Ptacin JL, Lee SF, Garner EC, Toro E, Eckart M, et al (2010) A spindle-like apparatus guides bacterial chromosome segregation. Nature Cell Biol 12: 791–798. 20657594 10.1038/ncb2083 PMC3205914

[29] Schofield WB, Lim HC, Jacobs-Wagner C (2010) Cell cycle coordination and regulation of bacterial chromosome segregation dynamics by polarly localized proteins. EMBO J 29: 3068–3081. 20802464 10.1038/emboj.2010.207 PMC2944072

[30] Fogel MA, Waldor MK (2006) A dynamic, mitotic-like mechanism for bacterial chromosome segregation. Genes Dev 20: 3269–3282. 17158745 10.1101/gad.1496506 PMC1686604

[31] Lutkenhaus J (2007) Assembly dynamics of the bacterial MinCDE system and spatial regulation of the Z ring. Annu Rev Biochem 76: 539–562. 17328675 10.1146/annurev.biochem.75.103004.142652

[32] Fu X, Shih YL, Zhang Y, Rothfield LI (2001) The MinE ring required for proper placement of the division site is a mobile structure that changes its cellular location during the *Escherichia coli* division cycle. Proc Natl Acad Sci USA 98: 980–985. 11158581 10.1073/pnas.031549298 PMC14695

[33] Raskin DM, de Boer PA (1999) Rapid pole-to-pole oscillation of a protein required for directing division to the middle of *Escherichia coli* . Proc Natl Acad Sci USA 96: 4971–4976. 10220403 10.1073/pnas.96.9.4971 PMC21801

[34] Hu Z, Lutkenhaus J (1999) Topological regulation of cell division in *Escherichia coli* involves rapid pole to pole oscillation of the division inhibitor MinC under the control of MinD and MinE. Mol Microbiol 34: 82–90. 10540287 10.1046/j.1365-2958.1999.01575.x

[35] Raskin DM, de Boer PA (1999) MinDE-dependent pole-to-pole oscillation of division inhibitor MinC in *Escherichia coli* . J Bacteriol 181: 6419–6424. 10515933 10.1128/jb.181.20.6419-6424.1999 PMC103778

[36] Hale CA, Meinhardt H, de Boer PA (2001) Dynamic localization cycle of the cell division regulator MinE in *Escherichia coli* . EMBO J 20: 1563–1572. 11285221 10.1093/emboj/20.7.1563 PMC145461

[37] Bi EF, Lutkenhaus J (1991) FtsZ ring structure associated with division in *Escherichia coli* . Nature 354: 161–164. 1944597 10.1038/354161a0

[38] Ma X, Ehrhardt DW, Margolin W (1996) Colocalization of cell division proteins FtsZ and FtsA to cytoskeletal structures in living *Escherichia coli* cells by using green fluorescent protein. Proc Natl Acad Sci USA 93: 12998–13003. 8917533 10.1073/pnas.93.23.12998 PMC24035

[39] Lutkenhaus J (2002) Dynamic proteins in bacteria. Curr Opin Microbiol 5: 548–552. 12457696 10.1016/s1369-5274(02)00376-4

[40] Rothfield L, Taghbalout A, Shih YL (2005) Spatial control of bacterial division-site placement. Nat Rev Microbiol 3: 959–968. 16322744 10.1038/nrmicro1290

[41] Hu Z, Lutkenhaus J (2000) Analysis of MinC reveals two independent domains involved in interaction with MinD and FtsZ. J Bacteriol 182: 3965–3971. 10869074 10.1128/jb.182.14.3965-3971.2000 PMC94581

[42] Dajkovic A, Lan G, Sun SX, Wirtz D, Lutkenhaus J (2008) MinC spatially controls bacterial cytokinesis by antagonizing the scaffolding function of FtsZ. Current Biol 18: 235–244. 10.1016/j.cub.2008.01.042 18291654

[43] Justice SS, García-Lara J, Rothfield LI (2000) Cell division inhibitors SulA and MinC/MinD block septum formation at different steps in the assembly of the *Escherichia coli* division machinery. Mol Microbiol 37: 410–423. 10931335 10.1046/j.1365-2958.2000.02007.x

[44] de Boer PA, Crossley RE, Rothfield LI (1992) Roles of MinC and MinD in the site-specific septation block mediated by the MinCDE system of *Escherichia coli* . J Bacteriol 174: 63–70. 1729224 10.1128/jb.174.1.63-70.1992 PMC205677

[45] Johnson JE, Lackner LL, de Boer PA (2002) Targeting of *^D^*MinC/MinD and *^D^*minC/DicB complexes to septal rings in *Escherichia coli* suggests a multistep mechanism for MinC-mediated destruction of nascent FtsZ rings. J Bacteriol 184: 2951–2962. 12003935 10.1128/JB.184.11.2951-2962.2002 PMC135045

[46] Hu Z, Gogol EP, Lutkenhaus J (2002) Dynamic assembly of MinD on phospholipid vesicles regulated by ATP and MinE. Proc Natl Acad Sci USA 99: 6761–6766. 11983867 10.1073/pnas.102059099 PMC124476

[47] Hu Z, Lutkenhaus J (2001) Topological regulation of cell division in *E. coli*: Spatiotemporal oscillation of MinD requires stimulation of its ATPase by MinE and phospholipid. Mol Cell 7: 1337–1343. 11430835 10.1016/s1097-2765(01)00273-8

[48] Howard M, Rutenberg AD, de Vet S (2001) Dynamic compartmentalization of bacteria: Accurate division in *E. coli* . Phys Rev Letters 87: 278102. 10.1103/PhysRevLett.87.278102 11800919

[49] Meinhardt H, de Boer PA (2001) Pattern formation in *Escherichia coli*: a model for the pole-to-pole oscillations of min proteins and the localization of the division site. Proc Natl Acad Sci USA 98: 14202–14207. 11734639 10.1073/pnas.251216598 PMC64659

[50] Kruse K (2002) A dynamic model for determining the middle of *Escherichia coli* . Biophys J 82: 618–627. 11806906 10.1016/S0006-3495(02)75426-X PMC1301873

[51] Huang KC, Meir Y, Wingreen NS (2003) Dynamic structures in *Escherichia coli*: Spontaneous formation of MinE rings and MinD polar zones. Proc Natl Acad Sci USA 100: 12724–12728. 14569005 10.1073/pnas.2135445100 PMC240685

[52] Drew DA, Osborn MJ, Rothfield LI (2005) A polymerization-depolymerization model that accurately generates the self-sustained oscillatory system involved in bacterial division site placement. Proc Natl Acad Sci USA 102: 6114–6118. 15840714 10.1073/pnas.0502037102 PMC1087953

[53] Meacci G, Ries J, Fischer-Friedrich E, Kahya N, Schwille P, et al (2006) Mobility of Min-proteins in *Escherichia coli* measured by fluorescence correlation spectroscopy. Phys Biol 3: 255–263. 17200601 10.1088/1478-3975/3/4/003

[54] Kerr RA, Levine H, Sejnowski TJ, Rappel WJ (2006) Division accuracy in a stochastic model of Min oscilations in *Escherichia coli* . Proc Natl Acad Sci USA 103: 347–352. 16387859 10.1073/pnas.0505825102 PMC1326155

[55] Fange D, Elf J (2006) Noise-induced Min phenotypes in *E. coli* . PLoS Comput Biol 2: e80. 16846247 10.1371/journal.pcbi.0020080 PMC1484588

[56] Tostevin F, Howard M (2005) A stochastic model of min oscillations in *Escherichia coli* and Min protein segregation during cell division. Phys Biol 3: 1–12. 16582457 10.1088/1478-3975/3/1/001

[57] Howard M, Kruse K (2005) Cellular organization by self-organization: mechanisms and models for Min protein dynamics. J Cell Biol 168: 533–536. 15716374 10.1083/jcb.200411122 PMC2171746

[58] Di Ventura B, Sourjik V (2011) Self-organized partitioning of dynamically localized proteins in bacterial cell division. Mol Systems Biol 7: 457. 10.1038/msb.2010.111 PMC3049411 21206490

[59] Arjunan SN, Tomita M (2010) A new multicompartmental reaction-diffusion modeling method links transient membrane attachment of *E. coli* MinE to E-ring formation. Syst Synth Biol 4: 35–53. 20012222 10.1007/s11693-009-9047-2 PMC2816228

[60] Bonny M, Fischer-Friedrich E, Loose M, Schwille P, Kruse K (2013) Membrane binding of MinE allows for a comprehensive description of Min-protein pattern formation. PLoS Comput Biol 9: e1003347. 24339757 10.1371/journal.pcbi.1003347 PMC3854456

[61] Woldringh CL, Mulder E, Valkenburg JA, Wientjes FB, Zaritsky A, et al (1990) Role of the nucleoid in the toporegulation of division. Res Microbiol 141: 39–49. 2194248 10.1016/0923-2508(90)90096-9

[62] Yu XC, Margolin W (1999) FtsZ ring cluster in *min* and partition mutants: Role of both the Min system and the nucleoid in regulating FtsZ ring localization. Mol Microbiol 32: 315–326. 10231488 10.1046/j.1365-2958.1999.01351.x

[63] Sun Q, Yu XC, Margolin W (1998) Assembly of the FtsZ ring at the central division site in the absence of the chromosome. Mol Microbiol 29: 491–503. 9720867 10.1046/j.1365-2958.1998.00942.x

[64] Bernhardt TG, de Boer PA (2005) SlmA, a nucleoid-associated, FtsZ binding protein required for blocking septal ring assembly over chromosomes in *Escherichia coli* . Mol Cell 18: 555–564. 15916962 10.1016/j.molcel.2005.04.012 PMC4428309

[65] Männik J, Wu F, Hol FJ, Bisicchia P, Sherratt DJ, et al (2012) Robustness and accuracy of cell division in *Escherichia coli* in diverse cell shapes. Proc Natl Acad Sci USA 109: 6957–6962. 22509007 10.1073/pnas.1120854109 PMC3345019

[66] Shih YL, Fu X, King GF, Le T, Rothfield L (2002) Division site placement in *E. coli* mutations that prevent formation of the MinE ring lead to loss of the normal midcell arrest of growth of polar MinD membrane domains. EMBO J 21: 3347–3357. 12093736 10.1093/emboj/cdf323 PMC126078

[67] Shih YL, Le T, Rothfield L (2003) Division site selection in *Escherichia coli* involves dynamic redistribution of Min proteins within coiled structures that extend between the two cell poles. Proc Natl Acad Sci USA 100: 7865–7870. 12766229 10.1073/pnas.1232225100 PMC164679

[68] Thanedar S, Margolin W (2004) FtsZ exhibites rapid movement and oscillation waves in helix-like patterns in *Escherichia coli* . Current Biol 14: 1167–1173. 10.1016/j.cub.2004.06.048 PMC4757587 15242613

[69] Anderson DE, Gueiros-Filho FJ, Erichson HP (2004) Assembly dynamics of FtsZ ring in *Bacillus subtilis* and *Escherichia coli* and effects of FtsZ-regulating proteins. J Bacteriol 186: 5775–5781. 15317782 10.1128/JB.186.17.5775-5781.2004 PMC516820

[70] Akerlund T, Bernander R, Nordström K (1992) Cell division in *Escherichia coli minB* mutants. Mol Microbiol 6: 2073–2083. 1406249 10.1111/j.1365-2958.1992.tb01380.x

[71] Teather RM, Collins JF, Donachie WD (1974) Quantal behavior of a diffusible factor which initiates septum formation at potential division sites in *Escherichia coli* . J Bacteriol 118: 407–413. 4597442 10.1128/jb.118.2.407-413.1974 PMC246772

[72] Marston AL, Thomaides HB, Edwards DH, Sharpe ME, Errington J (1998) Polar localization of MinD protein of *Bacillus subtilis* and its role in selection of the mid-cell division site. Genes Dev 12: 3419–3430. 9808628 10.1101/gad.12.21.3419 PMC317235

[73] Migocki MD, Freeman MK, Wake RG, Harry EJ (2002) The Min system is not required for precise placement of the midcell Z ring in *Bacillus subtilis* . EMBO Rep 3: 1163–1167. 12446561 10.1093/embo-reports/kvf233 PMC1308329

[74] Wery M, Woldringh CL, Rouviere-Yaniv J (2001) HU-GFP and DAPI co-localize on the *Escherichia coli* nucleoid. Biochimie 83: 193–200. 11278069 10.1016/s0300-9084(01)01254-8

[75] Bi EF, Lutkenhaus J (1990) FtsZ regulates frequency of cell division in *Escherichia coli* . J Bacteriol 172: 2765–2768. 2158979 10.1128/jb.172.5.2765-2768.1990 PMC208923

[76] Margolin W (2005) FtsZ and the division of prokaryotic cells and organelles. Nature Rev Mol Cell Biol 6: 862–871. 16227976 10.1038/nrm1745 PMC4757588

[77] Kruse K, Howard M, Margolin W (2007) An experimentalist's guide to computational modelling of the Min system. Mol Microbiol 63: 1279–1284. 17302810 10.1111/j.1365-2958.2007.05607.x PMC4758205

[78] Jaffé A, D'Ari R, Hiraga S (1988) Minicell-forming mutants of *Escherichia coli*: production of minicells and anucleate rods. J Bacteriol 170: 3094–3101. 2838458 10.1128/jb.170.7.3094-3101.1988 PMC211254

[79] Mulder E, EI'Bouhali M, Pas E, Woldringh CL (1990) The *Escherichia coli minB* mutation resembles *gyrB* in defective nucleoid segregation and decreased negative supercoiling of plasmids. Mol Gen Genet 221: 87–93. 2183010 10.1007/BF00280372

[80] Kerlund TA, Gullbrand B, Nordström K (2002) Effects of the Min system on nucleoid segregation in *Escherichia coli* . Microbiol 148: 3213–3222. 10.1099/00221287-148-10-3213 12368455

[81] Di Ventura B, Knecht B, Andreas H, Godinez WJ, Fritsche M, et al (2013) Chromosome segregation by the *Escherichia coli* Min system. Mol Systems Biol 9: 686. 10.1038/msb.2013.44 PMC3792344 24022004

[82] Fischer-Friedrich E, Friedrich BM, Gov NS (2012) FtsZ rings and helices: Physical mechanisms for the dynamic alignment of biopolymers in rod-shaped bacteria. Phys Biol 9: 016009. 22313630 10.1088/1478-3975/9/1/016009

[83] Sambrook J, Fritsch EF, Maniatis T (1989) Molecular cloning: a laboratory manual. Cold Spring Harbor Laboratory, Cold Spring Harbor, N. Y.

[84] Fried L, Lassak J, Jung K (2012) A comprehensive toolbox for the rapid construction of LacZ fusion reporters. J Microbiol Methods 91: 537–543. 23022912 10.1016/j.mimet.2012.09.023

[85] Chen JC, Beckwith J (2001) FtsQ, FtsL and FtsI require FtsK, but not FtsN, for co-localization with FtsZ during *Escherichia coli* cell division. Mol Microbiol 42: 395–413. 11703663 10.1046/j.1365-2958.2001.02640.x

[86] Bernhardt TG, de Boer PA (2003) The *Escherichia Coli* amidase AmiC is a periplasmic septal ring component exported via the twin-arginine transport pathway. Mol Microbiol 48: 1171–1182. 12787347 10.1046/j.1365-2958.2003.03511.x PMC4428285

[87] Miller VL, Mekalanos JJ (1988) A novel suicide vector and its use in construction of insertion mutation: Osmoregulation of outer membrane proteins and virulence determinants in *Vibrio cholerae* requires *toxR* . J Bacteriol 170: 2575–2583. 2836362 10.1128/jb.170.6.2575-2583.1988 PMC211174

[88] Thanbichler M, Iniesta AA, Shapiro L (2007) A comprehensive set of plasmids for vanillate- and xylose-inducible gene expression in *Caulobacter crescentus* . Nucl Acids Res 35: e137. 17959646 10.1093/nar/gkm818 PMC2175322

