# Supplementary material for: Effect of the Min System on Timing of Cell Division in Escherichia coli
Source: PLoS One. 2014 Aug 4;9(8):e103863. doi: 10.1371/journal.pone.0103863 (PMC4121188; doi:10.1371/journal.pone.0103863)
Supplement: File S1 — Combined file including theoretical background information and supporting figures. (PDF) [file pone.0103863.s001.pdf]

Supporting Information

Jia et al.

I. SI SUPPORTING FIGURES

Fig. S1: **OD plots.** WT (red) and *minB*$^-$ cells (blue) were grown in M9 medium with 0.5% glycerol and 1% Casimino acid at 30°C. Data are shown on a log scale.

Fig. S2: **OD curve as determined from the simulations.** We take total cell length as measure of OD of the culture. This quantity is calculated every minute in the simulations. As one can see the increase in total cell length is clearly linear on a log scale and the slope corresponds to a doubling time of 75 minutes.

Fig. S3: **The time dependence of the fraction of cells.** (a) Same as Fig. 3a, but data are obtained by averaging over two simulations (blue lines) respectively two experimental populations (red). (b) - (f) Individual curves of fractions with zero (b), one (c), two (d), three (e) and four chromosomes (f) with standard deviations as error bars. Simulations started with 7 cells.

Fig. S4: **Blocking of potential division sites.** In models 2-4 on average 2 of the potential division sites are blocked. In the example shown, the cell has 6 chromosomes and thus 7 potential division sites (5 non-polar, 2 polar sites), see (a). Out of these, 2 are (on average) blocked, indicated by red arrows in (b). Thus, chromosome aggregation occurs (on average) only at 5 out of the 7 potential division sites. These 5 sites are marked with green arrows in (c).

Fig. S5: **Results of model 2 for *minB*$^-$ cells.** (a) Cell length distribution and (b) cumulative distribution. As in Fig. 4 bars and dots represent experimental data. Solid red lines are the predictions of model 2. (c) Waiting time distribution of *minB*$^-$ cells for polar and non-polar sites. Solid lines are the results of model

2, bars represent experimental data (red: polar sites; blue: non-polar sites, see Fig. 4b). (d) Distribution and cumulative distribution of inter-division times for different types of cell division (same as Fig. 5 but for model 2). The cell division history of individual cells is summarized in (e), for details see Table 1.

Fig. S6: **Results of model 3 for *minB*$^-$ cells.** Same as Fig. S5 but for model 3.

II. SI THEORETICAL BACKGROUND: MODEL PARAMETERS

Here, we derive the details of cell growth and the parameter values that enter into the theoretical models.

A. Cell growth

To be able to implement cell growth in the simulations we needed to figure out if *E. coli* cells increase their mass (and thus length) exponentially or linearly with time (or in a more complicated way [1]). To do so we took pictures of the cells every 5 minutes and measured their lengths. We analyzed the data in two different ways to determine the increment in cell length per time.

1. *Rescaled cell length*

If cell length L increases exponentially with time t, then

$$L(t) = L_0 \exp[\frac{\ln 2}{T}(t - t_0)], \tag{1}$$

where L_0 is the newborn cell length (that can be different for different cells). T is the doubling time, and t_0 the time at which the cell was born. The last equation can be written as

$$\ln \frac{L}{L_0} = \frac{\ln 2}{T}(t - t_0), \tag{2}$$

where $\ln 2/T$ on the right hand side is a constant for the cells grown under the same condition. In particular, one obtains for the division length L_d

$$\ln \frac{L_d}{L_0} = \frac{\ln 2}{T}(t_d - t_0). \tag{3}$$

By combining Eq. (2) and Eq. (3) we can eliminate the difficulties caused by the differences in newborn cell length, i.e.

$$\frac{\ln L - \ln L_0}{\ln L_d - \ln L_0} = \frac{t - t_0}{t_d - t_0}. \tag{4}$$

Upon introducing the rescaled length increment

$$l_{resc} = \frac{\ln L - \ln L_0}{\ln L_d - \ln L_0}, \tag{5}$$

and the rescaled time

$$t_{resc} = \frac{t - t_0}{t_d - t_0}, \tag{6}$$

Eq. (4) then implies $l_{resc} = t_{resc}$, i.e. a linear correlation between cell length increment and time. Thus, for an exponential increase in cell mass all experimental data points should lie on a straight line from (0,0) to (1,1), see S7.

As can be seen, the rescaled experimental data (represented by blue dots) clearly lie on the straight line from (0,0) to (1,1). The red line shows how the curve would look like if the cells were increasing cell length linearly in time. In this way the differences between the two growth modes becomes apparent, indicating that the cells indeed increase their mass exponentially.

2. Cell length increment rate

Another way to distinguish exponential from linear mass increase is to calculate the cell length increment rate. For exponential time dependence, Eq. (1) implies

$$\frac{dL}{dt} = \frac{\ln 2}{T} L, \tag{7}$$

while for a linear increase one has

$$L = L_0 + \frac{L_d - L_0}{T} t, \tag{8}$$

and thus

$$\frac{dL}{dt} = \frac{L_d - L_0}{T}. \tag{9}$$

Eq. (7) shows that for an exponential mass increase the length increment rate is proportional to its length, while for a linear time dependence the length increment rate is constant.

From Fig. S8 we can see that for both strains the length increment rate is indeed proportional to the cell length, and the ratio is about the same for both strains. This is also consistent with our conclusion that these two strains have similar growth rates.

From the combination of these two methods, we conclude that the cells increase their length exponentially with time. In particular, from our experimental data we can also exclude the possibility of a linear or bilinear length (or mass) increase. In this case, in Fig. S8a all data points would line on or two horizontal lines.

B. Doubling time and division waiting time

In the simulations each cell gets a doubling time assigned that is drawn from the experimentally determined distribution of WT. We use the same distribution for *minB*$^-$ cells as the Min system does not affect cell growth, see OD plots in Fig. S1. The doubling time distribution shown in Fig. S9 was obtained by fitting the individual length increment curves of different cells with straight lines in a log scale plot.

As a control we determined the mean division waiting time in a different way. In Fig. S10 we show the number of cells together with the number of chromosomes in the population as function of time. Because chromosome duplication is synchronous with cell growth both quantities lie (in a log-linear scale) on two parallel

lines. The horizontal distance between them results from the phase separation of the growth and chromosome duplication cycles. Because cell division always happens after chromosome segregation in WT cells, the chromosome number is always larger than cell number. In fact, the distance between the two lines is the time difference between end of chromosome segregation and cell division, which is just the division waiting time. In this way, we obtained from Fig. S10 an average division waiting time of 17.7 minutes. As standard deviation we took 12 minutes as obtained from Fig. 2a.

The division waiting times of $minB^-$ cells were obtained by measuring the length increase of individual cells yielding the histogram shown in Fig. 2b. The distributions are different for non-polar (47±35.7min) and polar (76±34min) sites. One should note that because of the presence of mini cells (that do not contain chromosomes) cell number and chromosome number do not lie, as function of time, on parallel lines in a log-linear plot.

C. Cell lengths parameters

As mentioned above, in the simulations a starting length and an ending length are assigned to each new compartment. The ending length is twice as long as the starting length, but neither of them can be measured directly from the experimental data. What we can measure is the new born cell length of WT cells (Fig. S11). From the average new born cell length of WT L_0 we can calculate the starting length by

$$L_s = \frac{1}{2} L_0 \exp[\frac{\ln 2}{T}(T - T_w)]. \tag{10}$$

Here, T is the average doubling time in Fig. S9 and T_w is the waiting time in Fig. S10.

In the simulation the starting length is drawn from a normal distribution with average L_s and standard deviation $0.1 L_s$. This is set according to the distribution

of new born cell lengths (Fig. S11). Again, we used the length distribution of WT cells as a parameter for the $minB^-$ strain in the simulations.

---

[1] G. Reshes, S. Vanounou, I. Fishov and M. Feingold, Cell Shape Dynamics in *E. coli*, Biophys. J. **94**, 251 (2008).

III. SI FIGURE LEGENDS

Fig. S7: **Rescaled cell length increment as function of rescaled time.** The blue dots and error bars are experimental data. If cell mass increases exponentially with time the data points should lie on a straight line from (0,0) to (1,1). Data are for WT (a) and $minB^-$ cells (b). As a control, the rescaled curves for a linear cell length increment are shown in both figures as red lines.

Fig. S8: **The dependence of length increment rate on cell length.** Data are for WT (a) and for $minB^-$ cells (b).

Fig. S9: **The distribution of doubling times of WT.** 81 WT cells were measured. The average doubling time is 74min, the standard deviation is 17min.

Fig. S10: **Cell and chromosome number as function of time.** Cell number and chromosome number lie on parallel lines. The horizontal distance between them represents the division waiting time. Data are shown on a log-linear plot.

Fig. S11: **The distribution of cell length of newborn WT cells.** 81 cells were measured. The average cell length of newborn WT cells is 2.2μm. The distribution has a standard deviation of 0.31μm.

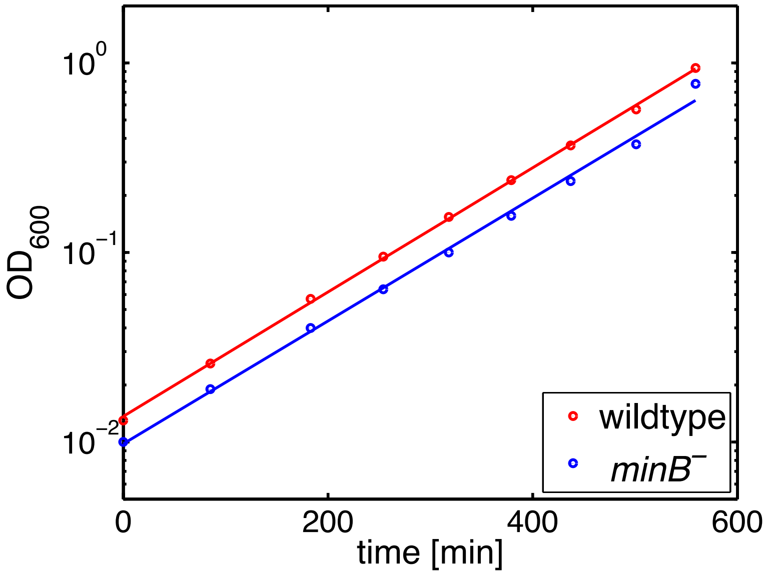

Figure S1

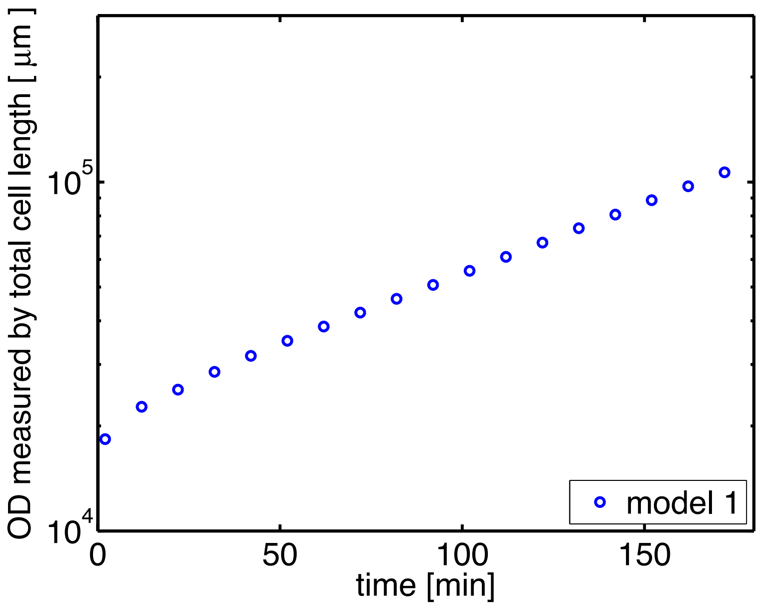

OD measured by total cell length [μm]
10^5
10^4
0
50
100
150
time [min]
model 1

Figure S2

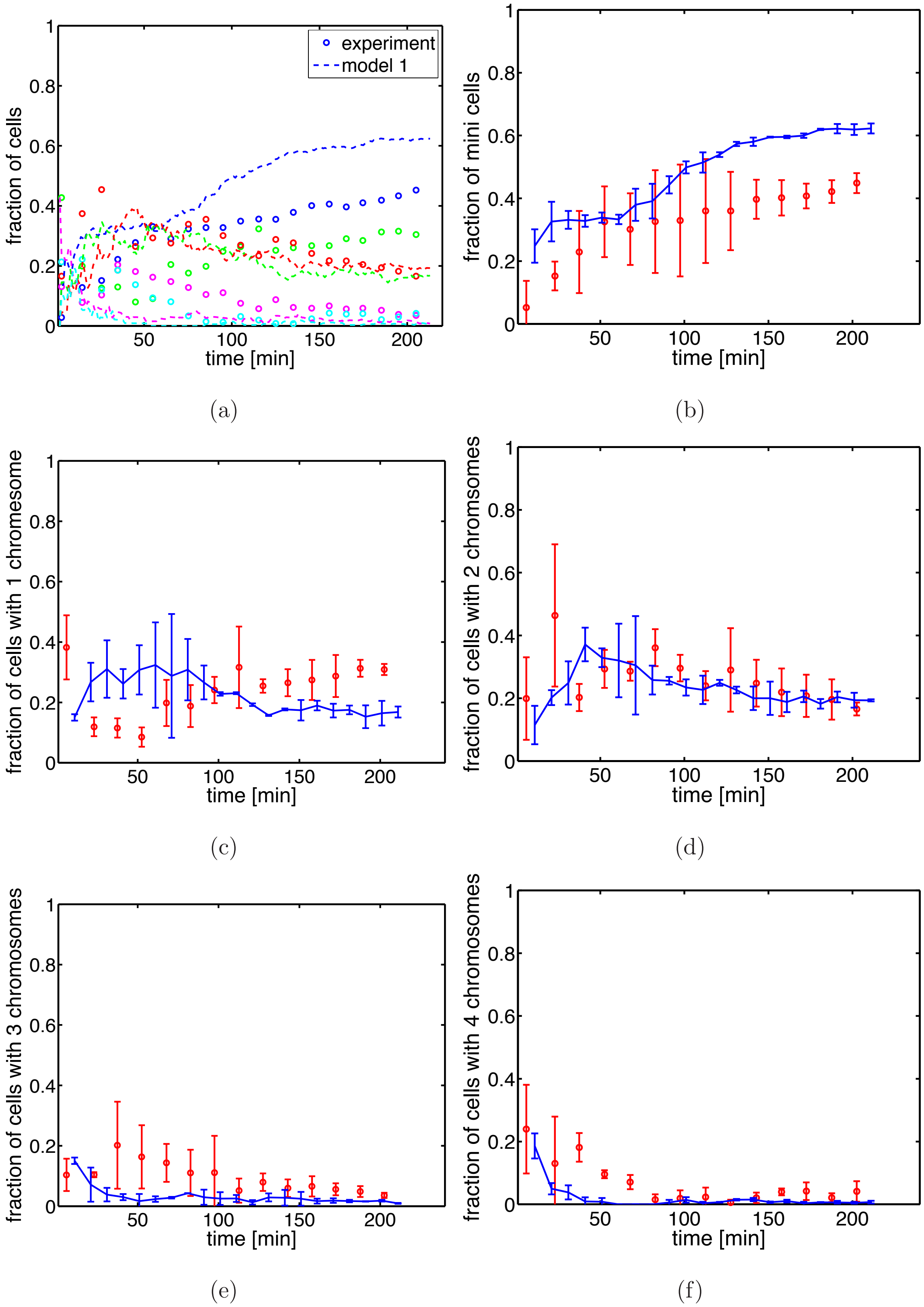

experiment
model 1
fraction of cells
fraction of mini cells
fraction of cells with 1 chromosome
fraction of cells with 2 chromosomes
fraction of cells with 3 chromosomes
fraction of cells with 4 chromosomes
time [min]
(a)
(b)
(c)
(d)
(e)
(f)

Figure S3

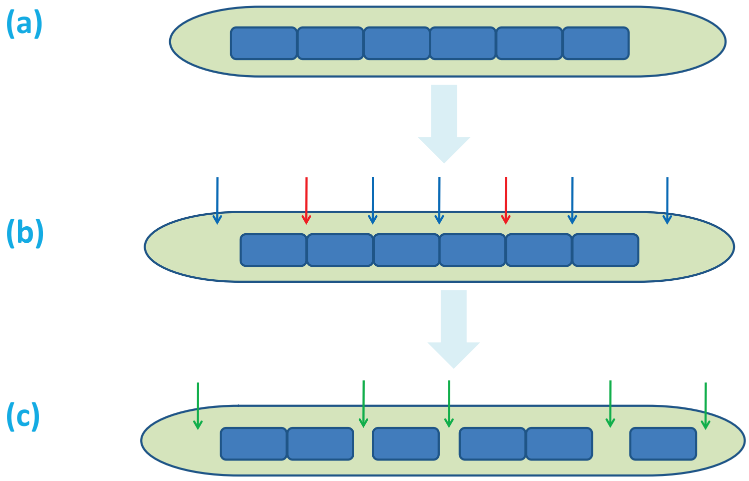

Figure S4

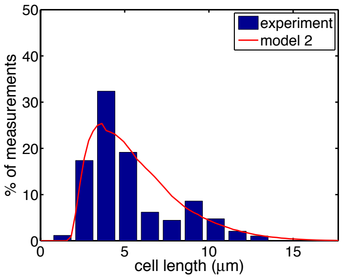

(a)

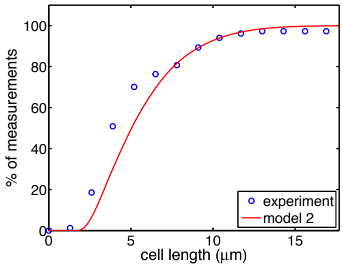

(b)

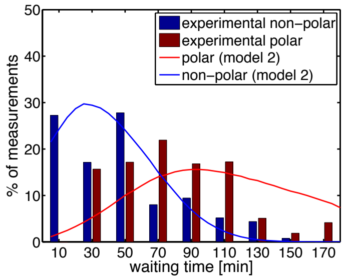

(c)

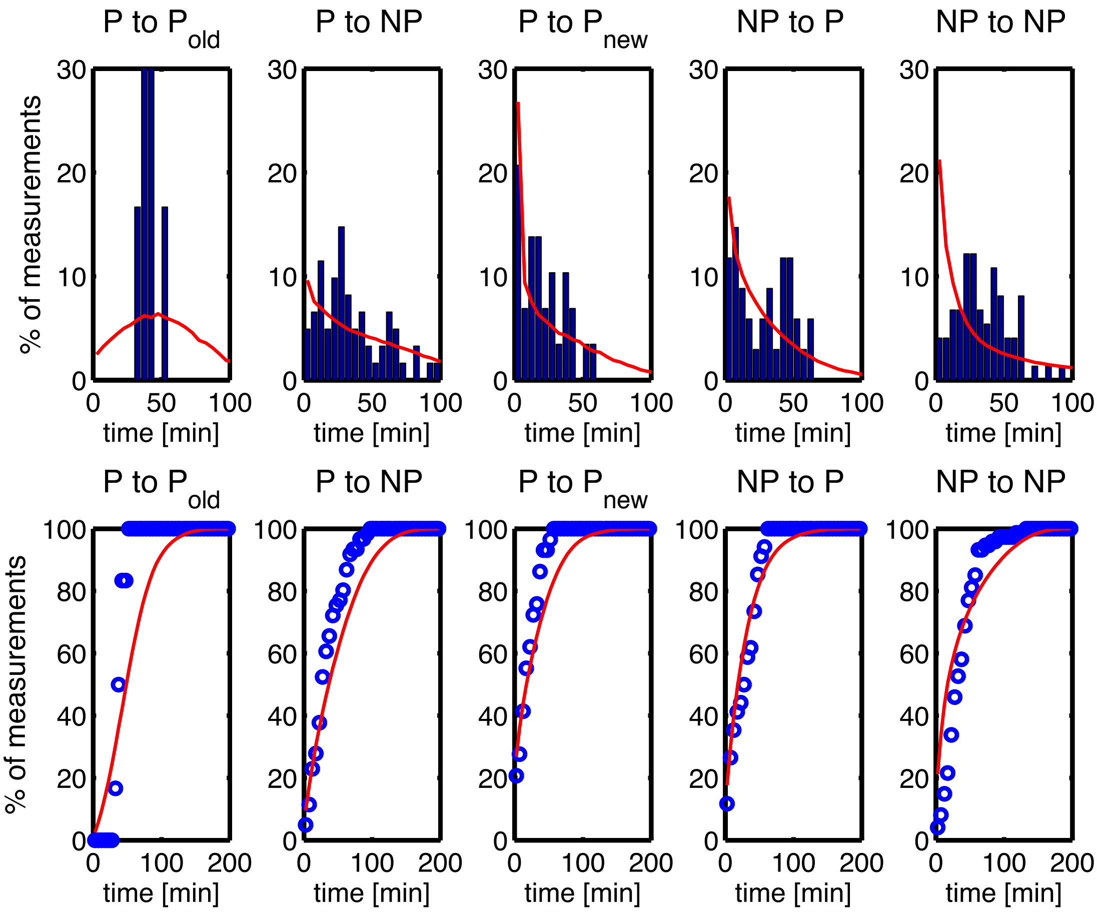

(d)

	%	old pole	non-polar	new pole
Experiment	polar	3 $(41.2 \pm 21.3\text{min})$	17 $(37 \pm 21.9\text{min})$	13 $(22.8 \pm 19.4\text{min})$
Experiment	non-polar	31 $(31.0 \pm 18.4\text{min})$	36 $(39.1 \pm 22.3\text{min})$	
Simulation	polar	2 $(55.2 \pm 32.3\text{min})$	19 $(48.1 \pm 38.3\text{min})$	6 $(30.4 \pm 31.2\text{min})$
Simulation	non-polar	33 $(39.8 \pm 28.5\text{min})$	40 $(36.2 \pm 39.3\text{min})$	

(e)

Figure S5

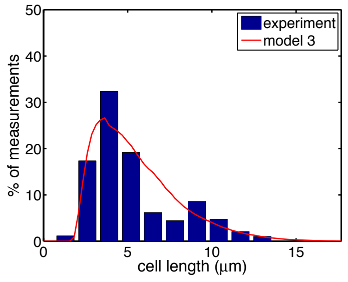

(a)

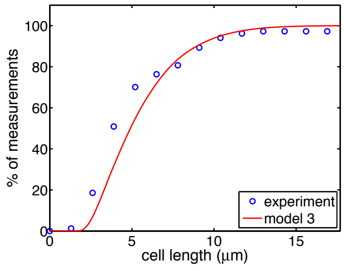

(b)

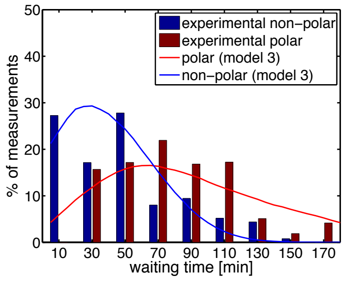

(c)

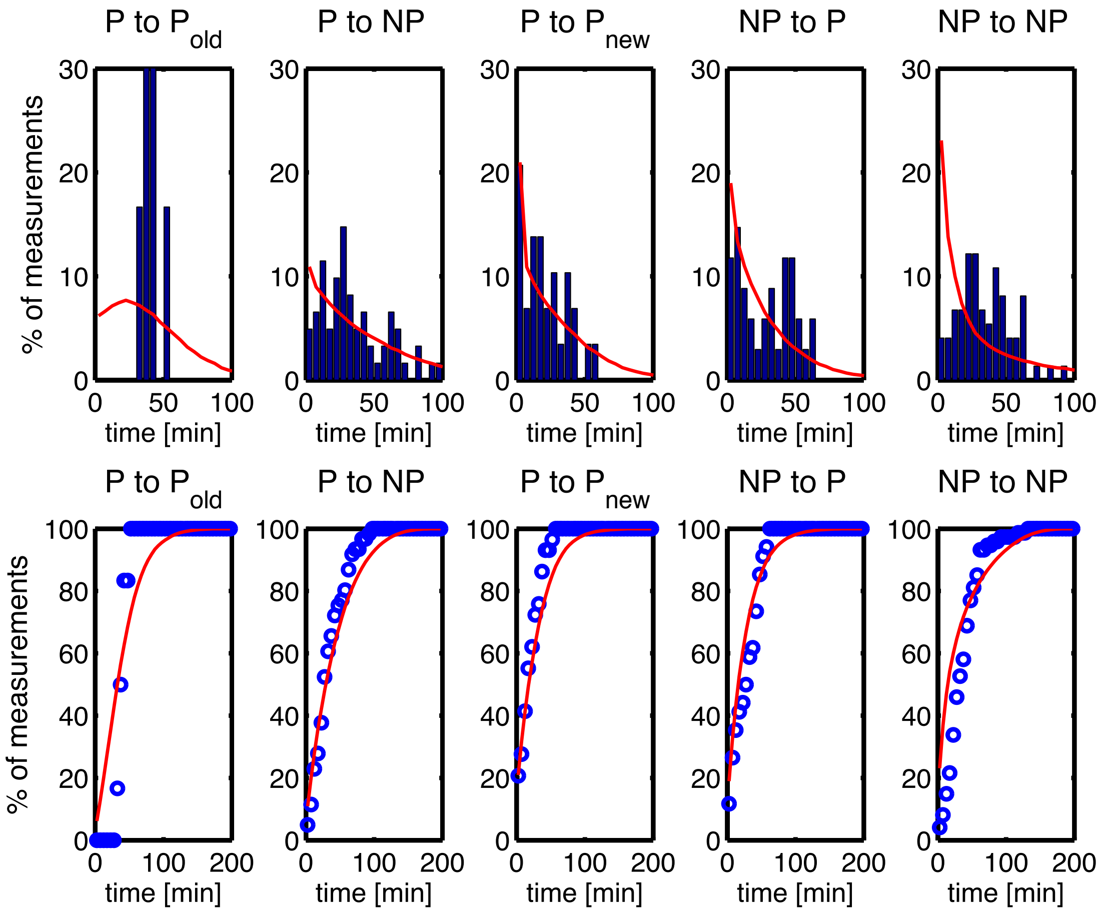

(d)

	%	old pole	non-polar	new pole
Experiment	polar	3 (41.2 ± 21.3min)	17 (37 ± 21.9min)	13 (22.8 ± 19.4min)
Experiment	non-polar	31 (31.0 ± 18.4min)	36 (39.1 ± 22.3min)	
Simulation	polar	5 (41.2 ± 29.3min)	20 (41.5 ± 35.1min)	10 (28.3 ± 26.8min)
Simulation	non-polar	35 (26.8 ± 25.8min)	40 (32.3 ± 36.2min)	

(e)

Figure S6

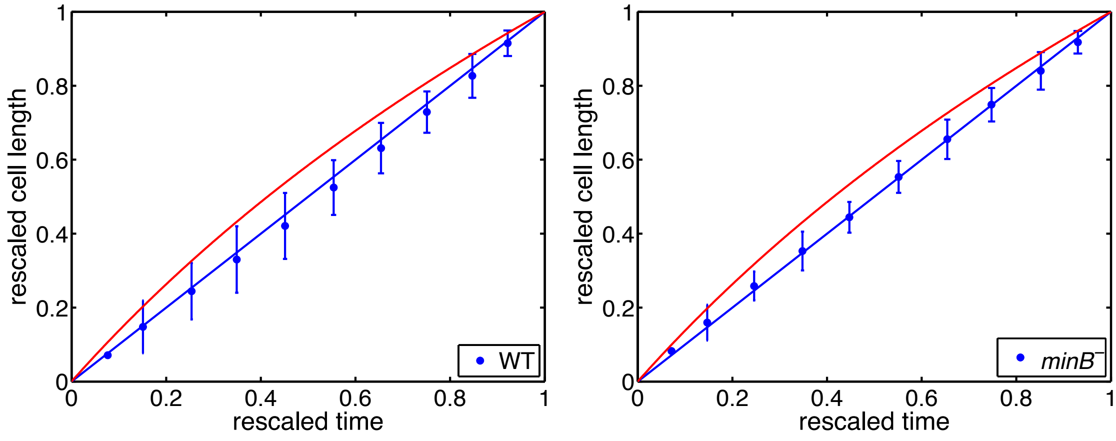
rescaled cell length
rescaled time
WT
rescaled cell length
rescaled time
minB⁻

(a) (b)

Figure S7

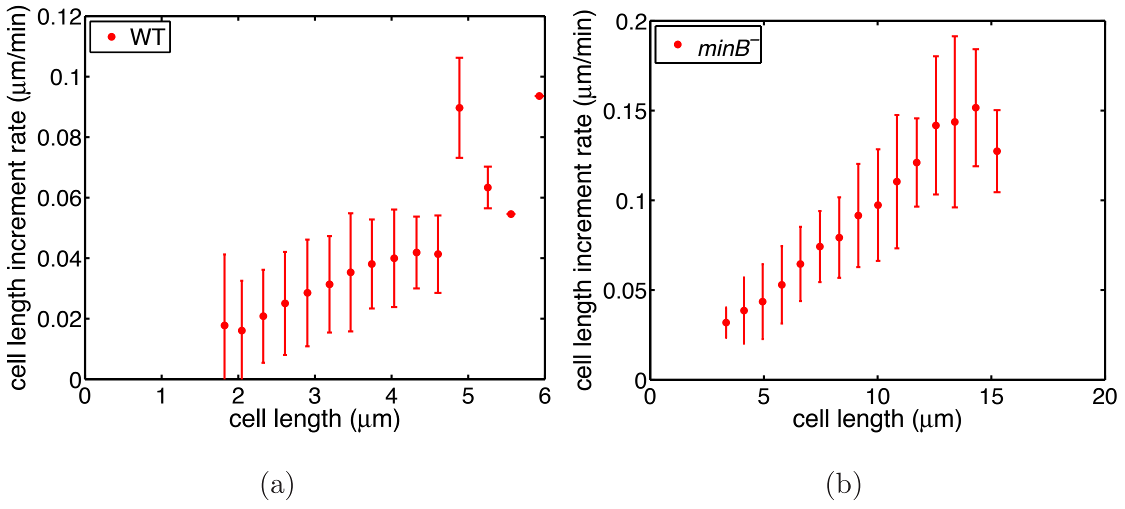

WT
cell length increment rate (μm/min)
cell length (μm)
0
0.02
0.04
0.06
0.08
0.1
0.12
0
1
2
3
4
5
6
(a)
minB⁻
cell length increment rate (μm/min)
cell length (μm)
0
0.05
0.1
0.15
0.2
0
5
10
15
20
(b)

Figure S8

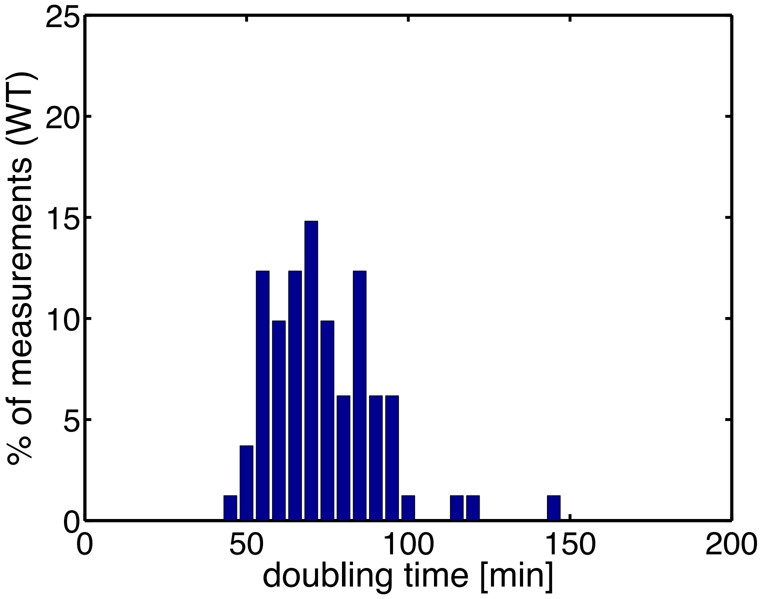

25
20
15
10
5
0
% of measurements (WT)
0
50
100
150
200
doubling time [min]

Figure S9

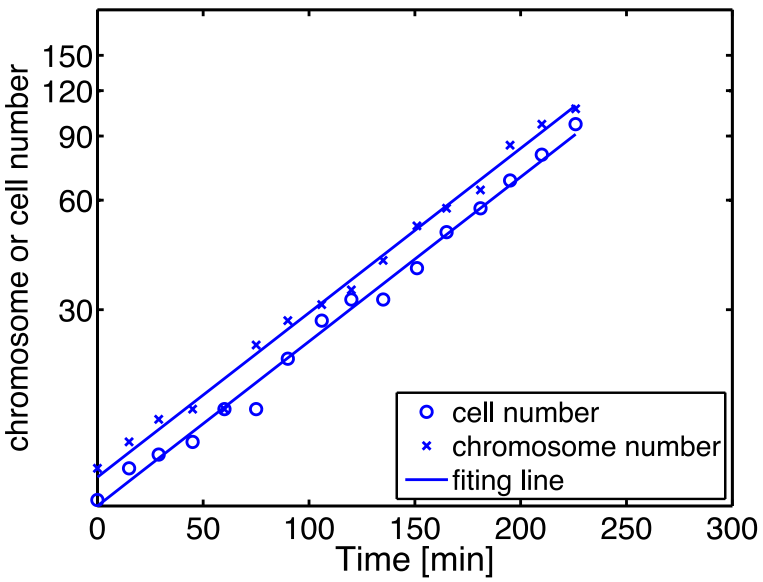
150
120
90
60
30
chromosome or cell number
0
50
100
150
200
250
300
Time [min]
cell number
chromosome number
fiting line

Figure S10

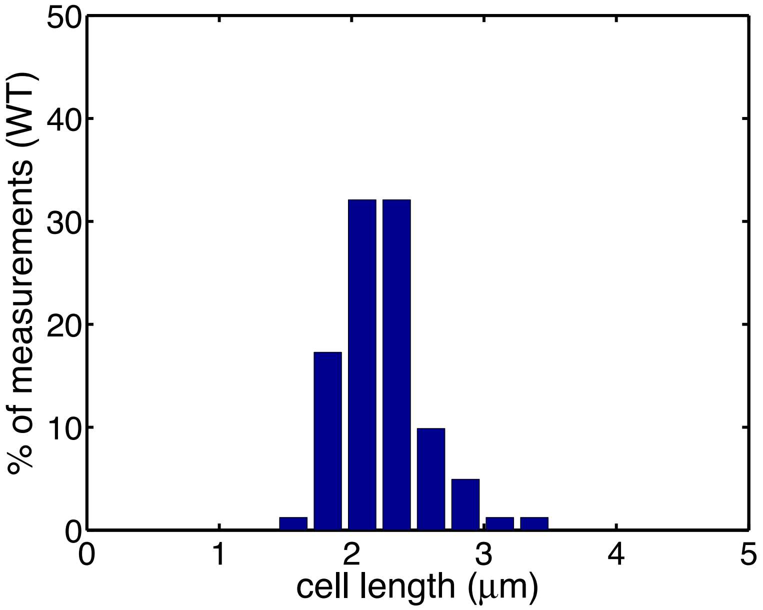

Figure S11
